# New genetic insights into HIV-associated neurocognitive disorder and Alzheimer's disease

**DOI:** 10.1016/j.gendis.2025.101576

**Published:** 2025-02-26

**Authors:** Hai Duc Nguyen, Woong-Ki Kim

**Affiliations:** a Division of Microbiology, Tulane National Primate Research Center, Tulane University, Covington, LA 70433, USA; b Department of Microbiology and Immunology, Tulane University School of Medicine, New Orleans, LA 70112, USA

HIV-associated neurocognitive disorder (HAND) is a central nervous system complication of HIV infection that affects cognitive, behavioral, and motor functions. The pathogenesis of HAND and its possible association with Alzheimer's disease (AD) remain unclear. This study used genomic data to reveal molecular mechanisms underlying HAND and key HAND biomarkers, with a focus on identifying new genetic variants, miRNAs, and transcription factors. We analyzed genomic studies, genome-wide association studies, and single-cell RNA sequencing datasets from cerebrospinal fluid and brain samples of individuals with HAND. Our objectives were to identify biomarkers associated with HAND and AD, validate them, and explore their interactions with genetic variants, miRNAs, and transcription factors. Our findings demonstrate significant decreases in synapse-related biomarkers and increases in immune system biomarkers in HAND. Key biomarkers, including *APOE*, *RHOA*, *DLG4*, *APP*, and *GAPDH*, were consistently altered across various datasets. Single nucleotide polymorphisms such as MTND4P3 [rs4718789-T], RNA5SP231 [rs4718789-T], and MSH6 [rs2098242-T] were identified as significant contributors to HAND pathogenesis, as were miRNAs hsa-miR-16-5p, hsa-miR-320a, and hsa-miR-335-5p. Transcription factors *THRA* and *NEUROD6* were also implicated in HAND. Altered expression of synapse-related and immune system biomarkers underscores the complex interplay between neurodegeneration and inflammation in HAND. The identified biomarkers and genetic variants offer potential avenues for further research and therapeutic development.

HAND impacts cognitive, behavioral, and motor functions in individuals with HIV/AIDS, reducing their quality of life.^1^ While combinational antiretroviral therapy (ART) has decreased severe dementia, milder forms of HAND still affect 30%–50% of patients on combinational ART. HAND shares some common features with AD, including neuroinflammation and certain genetic and miRNA markers, although the molecular links remain unclear.^2^ Genomic studies of HIV-infected individuals with HAND have identified potential biomarkers, but their impact is limited by small sample sizes, region coverage, and lack of focus on specific clinical stages of HAND and AD.^3^, ^4^, ^5^ The present study integrates genomic data and genome-wide association studies to identify key biomarkers in HAND and HIV-related AD as AD-like HIV-associated neurodegeneration, validates these findings with single-cell RNA sequencing, and maps interactions among biomarkers, miRNAs, and transcription factors to better understand HAND's pathogenesis and potential therapeutic targets (Fig. S1).

Eight studies analyzed gene expression in HIV-affected brain tissue, including cases with HAND, HIV-positive individuals with encephalitis (HIVE), and varying neurocognitive impairments (*e.g.*, mild neurocognitive disorder and normal cognition). Comparing 31 uninfected controls and 114 HIV-positive individuals, they examined brain regions such as the frontal cortex and neostriatum (Tables S1, S2). Altogether, 6925 gene expression changes were identified, with 3324 genes significantly altered in HIV-positive brains (log fold change >1.5, *P* < 0.05) (Fig. 1A).

**Figure 1:**
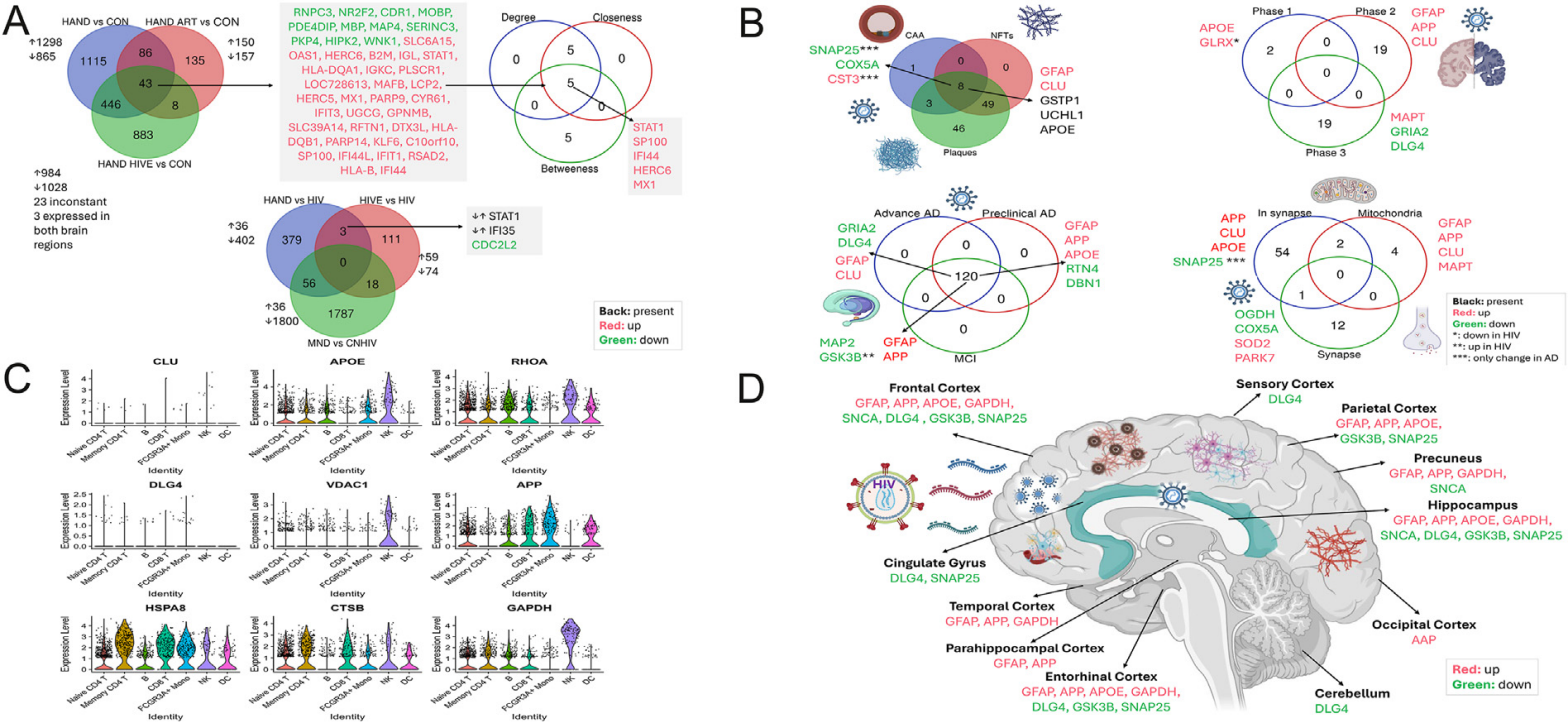
Differential gene expression profiles and molecular insights in HIV-associated neurocognitive disorder (HAND) brain tissues. **(A)** The Venn diagram showed shared and unique biomarkers among HAND versus healthy controls, HAND plus antiretroviral therapy (ART) versus healthy controls, and HAND plus HIVE versus healthy controls. This panel illustrates the shared and unique differentially expressed genes (DEGs) among various comparisons: HAND *vs*. controls (CON), HAND plus ART *vs*. CON, and HAND plus HIV encephalitis (HIVE) *vs*. CON. DEGs were identified based on an adjusted *P*-value < 0.05 (false discovery rate <5%) and log fold change (logFC) > ±0.58 (corresponding to fold change > 1.5). Notable biomarkers include STAT1, SP100, IFI44, HERC6, and MX1, with STAT1, CDC2L2, and IFI35 highlighted for their roles in HAND pathogenesis. Variations in biomarker expression reflect differences in tissue types and disease stages. HAND *vs*. CON: HIV-infected, ART-untreated individuals with HAND compared with uninfected controls; HAND ART *vs*. CON: HIV-infected, ART-treated individuals with HAND compared with uninfected controls; HAND HIVE *vs*. CON: HIV-infected, ART-untreated individuals with HAND and encephalitis compared with uninfected controls; HAND *vs*. HIV: ART-untreated individuals with HAND compared with HIV-infected, ART-untreated individuals; HIVE *vs*. HIV: ART-untreated individuals with encephalitis compared with HIV-infected, ART-untreated individuals; HIV plus MND *vs*. HIV: ART-untreated individuals with mild neurocognitive disorder (MND) compared with HIV-infected, ART-untreated individuals. **(B)** Analysis of important proteins implicated in Alzheimer's disease (AD) pathogenesis due to HIV. Centrality indicators were used to highlight proteins with significant roles in AD pathogenesis influenced by HIV infection, utilizing data from the NeuPro, GeneCards, and MalaCards databases. Proteins associated with key neuropathological hallmarks of AD, including synaptic dysfunction, mitochondrial abnormalities, and synapse-related proteins, are visualized. Differential expression analysis was performed using log fold change thresholds of > 1.5 and *P*-values < 0.05. Key proteins were identified using STRING v11.5 and edited in Cytoscape v3.9.1, applying a high-threshold score of 0.7 to filter protein–protein interactions. Network statistics, including degree, betweenness, and closeness centrality, were calculated using the CytoHubba application within Cytoscape. The top 10 genes based on centrality indicators were selected for further analysis. **(C)** Validation of HAND-related genes using single-cell RNA sequencing, showing gene expression in immune cell populations from HAND brain tissue samples. Single-cell RNA sequencing results validated HAND-related genes, showing their expression profiles in immune cell subpopulations from HAND brain tissue. Expression matrices were filtered to exclude cells with > 8% mitochondrial content, > 1.25% ATP content, or cells expressing < 500 or > 2000 genes. Differential abundance analysis identified significantly different subpopulations between HAND, HIV-positive, and healthy control groups. **(D)** Key AD-related proteins implicated in HIV, mapped to critical processes using multi-database cross-referencing. This panel highlights AD-related proteins implicated in HAND, identified through multi-database cross-referencing (GeneCards, MalaCards, and NeuPro). The proteins are associated with AD neuropathological features, including neurofibrillary tangles, amyloid plaques, and cerebral amyloid angiopathy. Differential expression analysis was performed using log fold change thresholds of > 1.5 and *P*-values < 0.05. Key proteins were identified using STRING v11.5 and edited in Cytoscape v3.9.1, applying a high-threshold score of 0.7 to filter protein–protein interactions. Network statistics, including degree, betweenness, and closeness centrality, were calculated using the CytoHubba application within Cytoscape. The top 10 genes based on centrality indicators were selected for further analysis.

Sixteen genes were differentially expressed in four or more studies, mainly in the frontal cortex. Key up-regulated genes included *B2M*, *HLA-C*, *IFI6*, and *MX1*, while *SYN2* was consistently down-regulated. Gene expression patterns varied by HIV condition (*e.g.*, HAND, HAND with encephalitis), revealing distinct molecular signatures for each neurocognitive subtype (Fig. S2).

HAND encompasses cognitive disorders in HIV-positive individuals, influenced by ART and HIVE. Our analysis identified 2163 biomarkers distinguishing HAND from controls and 307 ones specific to HAND with ART. Key biomarkers, including *STAT1*, *SP100*, *IFI44*, *HERC6*, and *MX1*, were shared across conditions and central in regulatory networks, highlighting their role in HAND pathogenesis. Significant gene expression differences across HAND subtypes and infected controls emphasize the involvement of immune and neuroinflammatory pathways (Fig. S3). Enrichment analysis revealed up-regulated immune and cytokine signaling pathways and reduced synaptic markers in HAND compared with controls, indicating synaptic and axonal disruptions. In ART-treated HAND, interferon signaling increased, while chromatin organization and translation-related proteins decreased. HAND plus HIVE cases without ART showed elevated significant immune activation and interferon signaling, and reduced neuronal projection proteins. Comparisons with HIV alone highlighted increased memory-impairment proteins and decreased synaptic function in HAND. HAND plus HIVE exhibited elevated interferon-related proteins, suggesting intensified immune responses with encephalitis (Fig. S3).

Given the overlap in neurodegenerative pathways between HIV neuroinfection and AD, we investigated potential connections using data from databases such as GeneCards and NeuroPro (Tables S3–S8). Among 262 shared genes implicated in both HAND and AD, seven key proteins — *APP*, *MAPT*, *AKT1*, *APOE*, *JUN*, *SNCA*, and *MAPK3* — were central to both diseases. Enrichment analysis demonstrated strong associations with synaptic and dopaminergic signaling, pathways central to both HAND and AD. Protein–protein interaction analysis further emphasized shared pathways related to neurodegeneration and synaptic transmission (Fig. S4–S6). We also found that these proteins were increased in both HIV-infected individuals and AD patients and were implicated in neuropathological features such as cerebral amyloid angiopathy, amyloid plaques, and neurofibrillary tangles (Fig. 1B).

To validate the relevance of 95 HAND-associated genes, we analyzed the single-cell RNA sequencing data from recent studies (GEO datasets GSE233717 and GSE202410) on brain tissue and cerebrospinal fluid samples from HIV-infected, ART-treated individuals with HAND. In HAND brain tissue, differential gene expression analysis revealed distinct gene patterns across immune cell types, with *APOE*, *RHOA*, *VDAC1*, and *APP* consistently up-regulated across cell populations (Fig. 1C; Fig. S7). In cerebrospinal fluid samples from ART-treated, HIV-positive individuals, *APOE*, *SNCA*, and *GSK3B* were among the significantly expressed genes (Fig. S8, S9). We also identified 95 HAND-associated genes that align with AD-related genes in the BrainSpan Atlas database (Table S9). These findings support the involvement of these genes in HAND-related neuroinflammatory and neurodegenerative processes across multiple immune cell types.

We examined gene regulatory networks involving transcription factors and miRNAs that modulate HAND-associated differentially expressed genes. Among the 95 key HAND-related genes, 39 were identified as interacting with specific miRNAs, highlighting their regulatory roles in HAND (Fig. S10 and Table S10). This analysis pinpointed 21 differentially expressed genes that are concurrently modulated by both miRNAs and transcription factors, underscoring the complexity of post-transcriptional regulation in HAND pathogenesis.

A total of 80 single nucleotide polymorphisms associated with HAND were identified across six GWAS databases (IDs: 28447399, 0007983, 22628157, 0007710, 0007982, 0002608) and mapped to various chromosomes (Table S11). Among these, the top 10 single nucleotide polymorphisms (*e.g.*, MTND4P3 (rs4718789-T), RNA5SP231 (rs4718789-T), MSH6 (rs2098242-T)), showed strong links to HAND pathogenesis. Enrichment analysis revealed associations with biological processes such as adaptive immune response and sodium ion transmembrane transport, consistent with prior studies (Fig. S11).

This study highlights the molecular mechanisms underlying HAND and HIV-related AD, as revealed through pathway enrichment and brain region-specific analyses. Immune-related pathways, such as interferon signaling and cytokine responses, were consistently up-regulated, while synaptic transmission and axonal function pathways were down-regulated, emphasizing the interplay between neuroinflammation and neurodegeneration in HAND. Brain region-specific analyses revealed distinct patterns: the frontal cortex and mid-frontal gyrus showed heightened immune activity with down-regulation of synaptic genes like *SYN2* (Fig. S2A); memory-related regions such as the hippocampus and entorhinal cortex exhibited impaired synaptic plasticity and stress-related gene expression (*e.g.*, *APOE*, *DLG4*, and *APP*); and areas like the neostriatum and precuneus displayed disruptions linking cognitive and motor impairments (*e.g.*, *SNCA* and *GAPDH*). Regions such as the parietal cortex and temporal cortex showed a mix of neuroinflammatory and synaptic dysfunction, while the cingulate gyrus highlighted behavioral and emotional regulation deficits (Fig. 1D). These findings underscore the region-specific complexity of HAND, with overlapping pathways shared with AD, particularly in memory and cognitive regions. This study provides a foundation for targeted interventions aimed at mitigating neuroinflammation and preserving synaptic integrity in HAND (Table S12). These findings underscore the importance of immune response and synaptic dysfunction in HAND, offering a molecular framework for understanding neurodegeneration in the context of HIV.

## CRediT authorship contribution statement

**Hai Duc Nguyen:** Writing – review & editing, Writing – original draft, Visualization, Validation, Supervision, Software, Resources, Project administration, Methodology, Investigation, Funding acquisition, Formal analysis, Data curation, Conceptualization. **Woong-Ki Kim:** Writing – review & editing, Validation, Supervision.

## Code availability

This manuscript did not produce any new code. All software or code used is open source, publicly accessible, or licensed. The methods explicitly detail any parameters used beyond the default settings.

## Data availability

The raw sequencing data for each research project can be obtained by following the instructions provided in the supplementary data. All further pertinent data that substantiates the conclusions of the research can be found in this publication and its supplementary information files or obtained from the corresponding author upon request.

## Funding

10.13039/100000002This work was supported by the National Institutes of Health Grant (R01MH118139 to W-KK) and also supported by the TNPRC P51 Base Grant (P51OD011104).

## Conflict of interests

The authors declared no competing interests.
