# Supplementary material for: New genetic insights into HIV-associated neurocognitive disorder and Alzheimer's disease
Source: Genes Dis. 2025 Feb 26;12(5):101576. doi: 10.1016/j.gendis.2025.101576 (PMC12142519; doi:10.1016/j.gendis.2025.101576)
Supplement: Multimedia component 1 [file mmc1.docx]

**Materials and methods**

**HAND databases**

We conducted a thorough literature search to identify all microarray studies of human brain tissue from individuals with HAND. Search results were automatically filtered using the Gene Expression Omnibus (GEO), European Nucleotide Archive (ENA), MEDLINE, Embase, Web of Science, and Google Scholar, with English language and human subject filters applied, using following search terms: ((((HIV) OR (human immunodeficiency virus)) OR (AIDS)) AND (((((Alzheimer's disease) OR (dementia)) OR (cognitive impairment)) OR (cognitive disorder)) OR (Neurocognitive disorder))) AND ((((microarray) OR (RNA-seq)) OR (transcriptomic)) OR (genetic profiling)). The search was limited to papers published up to August 8, 2024. We manually screened the results to select studies that met the following inclusion criteria: (1) studies that specifically examined differentially expressed genes (DEGs) in HAND brain tissue, and (2) investigations that explored the molecular processes linking HIV infection with neurocognitive disorders in brain tissue. HAND was defined according to the Frascati classification ^1^. Inclusion criteria for genomic studies were the analysis of human brain tissue, the use of microarray or RNA sequencing methods, and the availability of sufficient genomic data (DEGs, p-values, and fold-change). Genes were considered differentially expressed if they met certain statistical criteria, including FDR <5%, p <0.05 using the Kruskal-Wallis H-test, p <0.05 using ANOVA with an appropriate post hoc test (e.g., Tukey’s or Holm’s), or p <0.05 using a t-test combined with a fold change >1.5. Studies that did not specify brain regions or were purely in silico were excluded (Table 1). Data were exported from the HAND database for analysis (Supplementary Data 1). Each biomarker's HAND score (reflecting the number of times it was identified as DEG in various studies) was determined, with a maximum possible score of 8 (corresponding to 8 included studies). Biomarkers were classified as Up or Down in each study if they consistently showed the same directional change in at least 4 out of 8 studies (50%), with one exception allowed to account for variability across groups, drug use, brain regions, and disease stages. Biomarkers with two or more inconsistent directional changes were labeled as Inconsistent in the HAND database.

**Identify the association between Alzheimer’s disease and HIV infection**

To determine the association between AD and HIV infection, we identified shared genes using GeneCards and MalaCards ^2,3^. These databases offer detailed, user-friendly information on all annotated and predicted human genes, integrating data from approximately 75–150 web sources, including genetic, genomic, proteomic, transcriptomic, clinical, and functional data. A search was conducted using the keywords “HIV infection” and “Alzheimer’s disease” on August 8, 2024. We selected gene sets related to both HIV infection and AD from the “publication” tab and used Venn diagrams to visualize the overlap of shared genes. To validate our findings, we cross-referenced the shared genes with the HAND database and the NeuPro database, which provides information on protein changes in AD ^4^. We identified key biomarkers related to clinical stages of AD, specific brain regions, and neuropathological features, such as neurofibrillary tangles, amyloid plaques, and cerebral amyloid angiopathy, by comparing these biomarkers with the NeuPro database, and BrainSpan atlas (Supplementary Data 2, 3, 4, 5, 6, 7,8, 9). We also conducted a search for single-cell RNA sequencing (scRNA-seq) data in the GEO database (key terms: HIV, HAND, cognitive disorder, Alzheimer’s disease, scRNA-seq, single cell RNA sequencing) to further validate the key biomarkers associated with HAND pathogenesis using publicly available data from individuals with HAND.

Brain and CSF samples from both HIV-positive and HIV-negative control participants were processed using Seurat v3.0 and R version 3.4.2 ^5^. Gene-cell expression matrices from Cell Ranger were filtered by excluding: (i) cells with more than 8% mitochondrial transcript content; (ii) cells with over 1.25% ATP transcript content, and (iii) cells expressing fewer than 500 or more than 2,000 genes ^6^. The samples were then categorized and merged into separate groups: individuals with HAND, HIV-infected individuals without HAND, and healthy controls. For each merged dataset, we normalized library size, applied log transformation, performed scaling, selected variable features, and conducted principal component analysis (PCA). The top 20 principal components were used to compute t-distributed stochastic neighbor embedding (t-SNE) coordinates for visualization. Differential abundance analysis was conducted using DA-seq to identify the most significantly different cell subpopulations between various groups or tissues (Figure S1).

**Identify the association between SNPs and HAND**

The GWAS Catalog offers a comprehensive, searchable, and freely available database of SNP-trait associations available to scientists, clinicians, and researchers worldwide. As of July 27, 2024, the GWAS Catalog included 6,947 publications, 667,659 top associations, and 98,078 full summary statistics ^7^. For this study, we used the search terms specified above to investigate the potential of genetic variations as biomarkers for prognosis and therapeutic response in individuals diagnosed with HAND. Data was collected up to August 8, 2024. We manually reviewed the results to identify studies that met the predefined inclusion criteria, examining both reported traits and background traits. Data were excluded if they had missing beta values in the “Beta” tab, unclear variant or risk allele information, or p-values greater than 0.05 (Supplementary Data 10).

**Enrichment analysis**

Enrichment analyses, including biological processes, molecular functions, cellular components, and disease ontology, were conducted using Metascape, ToppGene Suite, and the ClueGO plug-in (version 2.5.8). Results with adjusted p-values < 0.05 were considered significant, with corrections made using the Benjamini-Hochberg method for multiple comparisons. For the ClueGO plug-in, signaling pathways were retrieved from KEGG, Reactome, and WikiPathways databases, and enrichment was assessed using a two-sided hypergeometric test with Bonferroni step-down correction. Terms were linked based on a κ score of 0.3. ClueGO integrates Gene Ontology (GO) terminology and KEGG/BioCarta pathways. Protein–protein interaction (PPI) networks were generated using STRING v11.5 and edited in Cytoscape v3.9.1, with a high-threshold score of 0.7 applied for filtering interactions ^8^. Network statistics, including degree, betweenness, and closeness, were calculated using the CytoHubba application within Cytoscape. To explore relationships between miRNAs and key biomarkers, we used the "MicroRNA Enrichment Turned Network" (MIENTURNET) ^9^. Functional enrichment analysis used Reactome, KEGG, WikiPathways databases, and disease ontology. A significance threshold of 0.05 was applied for functional annotations, with p-values adjusted using the Benjamini-Hochberg method.

We used miRTarBase to identify miRNAs experimentally verified to target our genes ^10^. miRTarBase provides experimentally validated miRNA-target interactions based on methods such as reporter assays, western blotting, quantitative polymerase chain reaction (qPCR), microarrays, and next-generation sequencing. These interactions are systematically curated from the literature to ensure accuracy (Supplementary Data 11). To further validate our findings, we conducted a search in the GEO database using terms such as “HAND,” “HIV infection,” “neurocognitive disorder,” and “miRNAs” to confirm the relevance of the identified miRNAs in the context of HAND. Transcription factors (TFs) responsible for gene expression alterations were identified using the ChEA3 tool ^11^. DEGs were input into ChEA3, which compared these gene sets with extensive TF target datasets using Fisher’s Exact Test with a reference size of 20,000. The results, including enrichment tables for each library and integration method, were used to construct and analyze gene, miRNA, and TF co-regulatory networks based on top findings using Cytoscape v3.9.1.

**References**

1. Antinori A, Arendt G, Becker JT, et al. Updated research nosology for HIV-associated neurocognitive disorders. *Neurology*. 2007;69(18):1789-1799. doi:10.1212/01.WNL.0000287431.88658.8b

2. GeneCards - Human Genes | Gene Database | Gene Search. Accessed August 8, 2024. https://www.genecards.org/

3. MalaCards - The Human Disease Database. Accessed August 8, 2024. https://www.malacards.org/

4. NeuroPro. Accessed August 8, 2024. https://neuropro.biomedical.hosting/

5. Stuart T, Butler A, Hoffman P, et al. Comprehensive Integration of Single-Cell Data. *Cell*. 2019;177(7):1888-1902.e21. doi:10.1016/j.cell.2019.05.031

6. Farhadian SF, Lindenbaum O, Zhao J, et al. HIV viral transcription and immune perturbations in the CNS of people with HIV despite ART. *JCI Insight*. 2022;7(13):e160267. doi:10.1172/jci.insight.160267

7. GWAS Catalog. Accessed August 8, 2024. https://www.ebi.ac.uk/gwas/docs/about

8. Nguyen HD, Kim WK, Huong Vu G. Molecular mechanisms implicated in protein changes in the Alzheimer’s disease human hippocampus. *Mech Ageing Dev*. 2024;219:111930. doi:10.1016/j.mad.2024.111930

9. Licursi V, Conte F, Fiscon G, Paci P. MIENTURNET: an interactive web tool for microRNA-target enrichment and network-based analysis. *BMC Bioinformatics*. 2019;20(1):545. doi:10.1186/s12859-019-3105-x

10. miRTarBase: the experimentally validated microRNA-target interactions database. Accessed August 9, 2024. https://mirtarbase.cuhk.edu.cn/~miRTarBase/miRTarBase_2022/php/index.php

11. Keenan AB, Torre D, Lachmann A, et al. ChEA3: transcription factor enrichment analysis by orthogonal omics integration. *Nucleic Acids Res*. 2019;47(W1):W212-W224. doi:10.1093/nar/gkz446

**Supplementary Figure legends**

**Figure S1 Detailed workflow for identifying and analyzing key genetic insights into HIV-associated neurocognitive disorder and Alzheimer's disease.**

**Figure S2 Differential gene expression profiles in HIV-infected brain tissues. (A)** Differentially expressed genes (DEGs) identified across studies comparing HIV^+^ HAND, HIV^+^ HIVE, HIV^+^ antiretroviral therapy (ART), and other groups with healthy controls. The figure shows the distribution and consistency of gene expression changes in the frontal cortex, with 16 genes identified as significantly altered in at least four studies. Genes such as B2M, HLA-C, IFI6, and MX1 exhibit consistent increases across all comparisons, while SYN2 shows a predominant decrease in HIV-positive brain tissues. Variability in directional changes is noted due to differences in disease stages, brain regions, and study groups. **(B)** The Venn diagram showed shared and unique biomarkers among HAND versus healthy controls, HAND plus ART versus healthy controls, and HAND plus HIVE versus healthy controls. STAT1, SP100, IFI44, HERC6, and MX1 are highlighted for their central role in HAND pathogenesis, whereas STAT1, CDC2L2, and IFI35 are highlighted for their central role in HAND pathogenesis when compared with HIV-infected individuals. Inconsistencies in biomarker expression across studies are illustrated, reflecting variations in tissue types and disease stages. HIV: human immunodeficiency virus; HAND, HIV-associated neurocognitive disorder; HIVE: HIV encephalitis; HIV plus MND: HIV position with mild neurocognitive disorder (MND); HIV-positive NC: HIV position with normal cognition; Controls: uninfected controls. HAND *vs*. CON: HIV-infected HAND cases untreated with ART versus uninfected controls; HAND ART *vs*. CON: HIV-infected HAND cases treated with ART versus uninfected controls; HAND HIVE *vs*. CON: HIV-infected HAND plus encephalitis cases untreated with ART versus uninfected controls; HAND *vs*. HIV: HIV infected HAND cases untreated with ART versus HIV infected cases untreated with ART; HIVE *vs*. HIV: HIV infected encephalitis cases untreated with ART versus HIV infected cases untreated with ART; HIV plus MND *vs*. HIV: HIV infected MND cases untreated with ART versus HIV infected cases untreated with ART.

**Figure S3 Molecular mechanisms and cellular components in** HIV-associated neurocognitive disorder (**HAND) and related conditions. (A)** Enrichment analysis of biological processes and cellular components in HAND versus healthy controls. Increased activity in innate immune response and cytokine signaling is observed, alongside a decrease in proteins related to synaptic function and axonal integrity. **(B)** HAND plus antiretroviral therapy (ART) versus healthy controls, showing increased expression of viral response and interferon signaling proteins and decreased expression of proteins related to chromatin organization and translation. **(C)** HAND plus HIV encephalitis (HIVE) versus healthy controls, illustrating significant increases in viral response and interferon signaling proteins and reductions in synaptic and axonal proteins. **(D)** HAND versus HIV, highlighting increased levels of memory impairment-related proteins and decreased levels of proteins involved in glutamine metabolism and synaptic functions. **(E)** HAND plus HIVE versus HIV, showing increased interferon signaling proteins and decreased axonal and synaptic proteins. **(F)** Mild neurocognitive disorder (MND) versus HIV-positive NC, with increased levels of proteins associated with membrane trafficking and axon guidance, and decreased nucleolus function proteins. Key processes, including synaptic transmission and dopaminergic signaling pathways, were mapped using databases such as KEGG and Reactome, emphasizing their relevance to both HAND and Alzheimer's disease pathogenesis. HAND *vs*. HIV: HIV-infected HAND cases untreated with ART versus HIV-infected cases untreated with ART. Statistical thresholds (*P* < 0.05, Benjamini-Hochberg correction) were applied to identify significant overlaps.

**Figure S4 Association between HIV infection and Alzheimer's disease (AD). (A, B)** Validation of shared genes between HIV and AD using the GeneCards and MalaCards databases. 262 genes implicated in AD pathogenesis related to HIV infection are visualized. **(C–E)** Enrichment analysis of proteins associated with specific signaling pathways, including synaptic transmission and dopaminergic signaling, which are relevant to AD. **(F)** Identification of key proteins (APP, MAPT, MAPK3, AKT1, APOE, JUN, SNCA) using centrality indicators. **(G)** Expression levels of these proteins in HIV-infected brains and their association with AD neuropathological features such as amyloid plaques and neurofibrillary tangles. These proteins were validated using the NeuPro database as well as manual research on Pubmed. Data were analyzed using enrichment tools (Metascape, ToppGene Suite), and results with adjusted *P*-value <0.05 were considered significant. The comparisons of the reduction of synaptic function proteins in HIV-associated neurocognitive disorder (HAND) versus HIV highlight the neurocognitive impact of HAND.

**Figure S5 Analysis of important proteins implicated in** Alzheimer's disease **pathogenesis due to HIV.** **(A)** Central indicators highlight proteins with significant roles in Alzheimer's disease using the NeuPro, GeneCards, and MalaCards databases. **(B–D)** Visualization of proteins associated with key neuropathological hallmarks of Alzheimer's disease, including synaptic proteins, mitochondrial proteins, and those at synapses.

**Figure S6 Cross-referencing data from multiple databases for** Alzheimer's disease**-related proteins. (A)** Identification of 95 shared genes across HIV-associated neurocognitive disorders, using the NeuroPro, GeneCard, and MalaCards databases. Important proteins such as APOE, SNCA, DLG4, and GAPDH are highlighted. **(B–D)** Protein–protein interaction enrichment analysis revealed key pathways related to Alzheimer's disease and neurodegeneration. **(E)** Proposed key proteins implicated in Alzheimer's disease pathogenesis due to HIV infection, visualized with connections to critical processes.

**Figure S7 Validation of** HIV-associated neurocognitive disorder (**HAND)-related genes using single-cell RNA sequencing. (A)** Differentially expressed genes (DEGs) in HAND brain tissues from the GSE233717 dataset, showing expression patterns across different cell types. **(B)** Key DEGs identified with central indicators. **(C, D)** Expression of HAND-related genes in various immune cell populations within HAND brain tissues. Expression matrices were filtered to exclude cells with >8% mitochondrial content, >1.25% ATP content, or cells expressing <500 or >2000 genes. Differential abundance analysis identified significantly different subpopulations between HAND, HIV-positive, and healthy control groups.

**Figure S8 Validation of** HIV-associated neurocognitive disorder (**HAND)-related genes in** cerebrospinal fluid (**CSF) samples using** single-cell RNA sequencing**. (A)** Differentially expressed genes (DEGs) identified in HIV-infected CSF samples from the GSE202410 dataset, with significant expression of genes in immune cell populations. **(B)** Key DEGs with central indicators. **(C, D)** Expression patterns of HAND-related genes in different immune cells within HIV-infected and healthy CSF samples. Expression matrices were filtered to exclude cells with >8% mitochondrial content, >1.25% ATP content, or cells expressing <500 or >2000 genes. Differential abundance analysis identified significantly different subpopulations between HAND, HIV-positive, and healthy control groups.

**Figure S9 Comparison of** differentially expressed genes (**DEGs) between HIV-infected and healthy** cerebrospinal fluid (**CSF) samples. (A)** DEGs in CSF samples from healthy controls and HIV-infected individuals, with central indicators highlighting key DEGs. **(B)** Expression of HIV-associated neurocognitive disorder (HAND)-related DEGs across immune cell populations in healthy CSF samples. Expression matrices were filtered to exclude cells with >8% mitochondrial content, >1.25% ATP content, or cells expressing <500 or >2000 genes. Differential abundance analysis identified significantly different subpopulations between HAND, HIV-positive, and healthy control groups.

**Figure S10 The regulatory network of** differentially expressed genes (**DEGs), miRNAs, and transcription factors (TFs). (A)** The DEG-miRNA-TF interactions mapped using miRTarBase. **(B)** Network visualization with circular nodes for DEGs, diamond-shaped nodes for TFs, and rectangular nodes for miRNAs. **(C, D)** Significant miRNAs involved in Alzheimer's disease-related signaling pathways. **(E)** TF co-expression network showing activity in different brain regions, with notable TFs such as THRA and NEUROD6. The network was constructed using ChEA3, miRTarBase, and Cytoscape, with top regulators selected based on Fisher's exact test (*P* < 0.05) and visualization of interactions above a STRING confidence score of 0.7.

**Figure S11** Single nucleotide polymorphisms (**SNPs) associated with** HIV-associated neurocognitive disorder (**HAND). (A)** Distribution of 80 SNPs associated with HAND across chromosomes. **(B)** Ten SNPs with the lowest *P*-values and highest beta or odds ratios, including MTND4P3 and MSH6, highlighted for their role in HAND pathogenesis. **(C, D)** Enrichment analysis showed significant associations of these SNPs with various biological processes and molecular functions relevant to HAND. A figure highlights the top 10 SNPs associated with HAND, based on GWAS data (*P* < 0.05). SNPs with the lowest *P*-values and highest beta or odds ratios include MTND4P3 and MSH6, underscoring their significance in HAND pathogenesis. Confidence intervals and effect sizes for these associations are provided.

**Supplementary Table legends**

**Table S1** The studies included in the HIV-associated neurocognitive disorder database.

**Table S2** The HIV-associated neurocognitive disorder database (Biomarkers changes involved in the pathogenesis of Alzheimer's disease caused by HIV).

**Table S3** The biomarkers associated with Alzheimer's disease.

**Table S4** The biomarkers associated with HIV.

**Table S5** The overlapped biomarkers associated with HIV and Alzheimer's disease.

**Table S6** The association between overlapped biomarkers (genes related to HIV and dementia) and the NeuroPro database involved in the pathogenesis of Alzheimer's disease.

**Table S7** The association between overlapped biomarkers (genes related to HIV and Alzheimer's disease) and the NeuroPro database involved in different clinical stages of Alzheimer's disease.

**Table S8** The key overlapped biomarkers changed in HIV brains.

**Table S9** Validation of miRNAs experimentally to target 95 HIV-associated neurocognitive disorder-associated genes using the BRAINSPAN dataset (https://www.brainspan.org/static/download.html).

**Table S10** Validation of miRNAs experimentally to target 95 HIV-associated neurocognitive disorder-associated genes using miRTarBase.

**Table S11** Combined human genome-wide association studies implicated in the pathogenesis of Alzheimer's disease caused by HIV.

**Table S12** Summary of key findings from differential gene expression analysis in HIV-associated neuroinflammation and HIV-associated neurocognitive disorder: gene identification, pathways, and validation across studies.
