# Supplementary material for: New genetic insights into HIV-associated neurocognitive disorder and Alzheimer's disease
Source: Genes Dis. 2025 Feb 26;12(5):101576. doi: 10.1016/j.gendis.2025.101576 (PMC12142519; doi:10.1016/j.gendis.2025.101576)
Supplement: Multimedia component 2 [file mmc2.docx]

**Table S1.** Studies included in HAND database

| **Authors (year)** | **Study design** | **Groups** | **Brain area** | **DEGs** | **Methods** | **Statistical approach** | **References** |
| --- | --- | --- | --- | --- | --- | --- | --- |
| Masliah et al. 2004 | - HIV+ with HIVE (n= 9) vs. HIV+ no HIVE (n=8) | - HIV+ - HIVE | - Frontal cortex | 133 | - TRIzol Reagent - Affymetrix Test 2 chips - Microarray | FC >1.5 and p < 0.05 | (Masliah *et al.*, 2004) |
| Gelman et al. 2004 | - HIV+ with HAND (n = 9) vs. healthy controls (n = 5) | - HIV+ HAND - Uninfected controls | - Frontal cortical grey matter - Adjacent gyral white matter | 18 | - Acid guanidinium thiocyanate-phenol-chloroform extraction - Affymetrix HG-U133 chips - Microarray | p < 0.05 | (Gelman *et al.*, 2004) |
| Everall et al. 2005 | - HIV+ (n = 5 incl. HIVE (n = 3) vs. healthy controls (n= 4) - HAD (n = 3)) vs. healthy controls (n= 4) - HIV+ (n = 5 incl. HIVE (n = 3) and HAD (n = 3)) vs. healthy controls (n= 4) | - HIVE - HIV+ HAND - Uninfected controls | - Frontal cortex | 130 | - TRIzol Reagent - Affymetrix Test 2 chips - Microarray | p < 0.05 | (Everall *et al.*, 2005) |
| Borjabad et al. 2011 | - HAND (n=7) vs. healthy controls (n=6) - HIV+ HAND (n=8) vs. HIV+ HAND cART (n=7) | - HIV+ HAND - HIV+ HAND ART - Uninfected controls | - Deep white matter (anterior frontal lobe) | 2240 | - RNeasy Mini Kit (Qiagen) - Affymetrix Human Genome 133 plus 2.0 arrays - Microarray | FC >1.5 and p < 0.05 | (Borjabad *et al.*, 2011) |
| Gelman et al. 2012 | - HIV+ with neurocognitive impairment and HIVE (n=5) vs. HIV+ with NCI, no HIVE (n=7) vs. Uninfected controls (n=6) - HIV+ with neurocognitive impairment (n=5) vs. Uninfected controls (n=6) - HIV+ with NCI, no HIVE (n=7) vs. HIV+/CN, no HIVE (n=6) vs. Uninfected controls (n=6) | - No HIVE - HIVE - HIV+ HIVE - Uninfected controls | - Frontal cortex, white matter - Frontal cortex, neostriatum - Neostriatum, white matter | 1989 | - TRIzol Reagent - Affymetrix Human Genome 133 plus 2.0 arrays - Microarray | p < 0.05  and  Benjamini and Hochberg adjusted p<0.05 | (Gelman *et al.*, 2012) |
| Zhou et al. 2012 | - HIV+ with HAD (n=10) vs. HIV+ no HAD (n=8) | - HIV+ HAND - HIV+ no HAND | - Frontal Cortex | 438 | - Qiagen miRNeasy Mini kit- Qiagen - Agilent 2100 Bioanalyser - Microarray | p < 0.05 | (Zhou *et al.*, 2012) |
| Canchi et al. 2020 | - HIV+ with HAD (n = 3) vs. HIV+ with MCD (n = 10) vs. HIV+ with asymptomatic neurocognitive impairment (n=10) vs. HIV+/cognitive normal (n=10) | - HIV HAND - HIV + no HAND | - Frontal Cortex | 1861 | - RNeasy plus mini kit (Qiagen) - Agilent 2100 Bioanalyser - RNAseq (75 bp single end reads with coverage of 20 million) | p < 0.05  and  FDR<0.05 | (Canchi *et al.*, 2020) |
| Mohammadzadeh et al.2023 | - HIV+/HAND (n  =  10) vs. Uninfected controls (n  =  10) | - HIV+ HAND - Uninfected controls | - Mid-frontal gyrus | 116 | - TRIzol Reagent - RNAseq | p < 0.05 | (Mohammadzadeh *et al.*, 2023) |

HIV: human immunodeficiency virus; HAD: HIV-associated dementia; HIVE: HIV encephalitis; DEGs: differentially expressed genes; FDR: false discovery rate; MND: HIV+ with mild cognitive disorder; NCI: normal cognitive impairment.

**References**

Borjabad, A. *et al.* (2011) ‘Significant Effects of Antiretroviral Therapy on Global Gene Expression in Brain Tissues of Patients with HIV-1-Associated Neurocognitive Disorders’, *PLoS Pathogens*, 7(9), p. e1002213. Available at: https://doi.org/10.1371/journal.ppat.1002213.

Canchi, S. *et al.* (2020) ‘Transcriptomic analysis of brain tissues identifies a role for CCAAT enhancer binding protein β in HIV-associated neurocognitive disorder’, *Journal of Neuroinflammation*, 17(1), p. 112. Available at: https://doi.org/10.1186/s12974-020-01781-w.

Everall, I. *et al.* (2005) ‘Methamphetamine stimulates interferon inducible genes in HIV infected brain’, *Journal of Neuroimmunology*, 170(1), pp. 158–171. Available at: https://doi.org/10.1016/j.jneuroim.2005.09.009.

Gelman, B.B. *et al.* (2004) ‘Acquired neuronal channelopathies in HIV-associated dementia’, *Journal of Neuroimmunology*, 157(1), pp. 111–119. Available at: https://doi.org/10.1016/j.jneuroim.2004.08.044.

Gelman, B.B. *et al.* (2012) ‘The National NeuroAIDS Tissue Consortium brain gene array: two types of HIV-associated neurocognitive impairment’, *PloS One*, 7(9), p. e46178. Available at: https://doi.org/10.1371/journal.pone.0046178.

Masliah, E. *et al.* (2004) ‘Patterns of gene dysregulation in the frontal cortex of patients with HIV encephalitis’, *Journal of Neuroimmunology*, 157(1), pp. 163–175. Available at: https://doi.org/10.1016/j.jneuroim.2004.08.026.

Mohammadzadeh, N. *et al.* (2023) ‘The HIV Restriction Factor Profile in the Brain Is Associated with the Clinical Status and Viral Quantities’, *Viruses*, 15(2). Available at: https://doi.org/10.3390/v15020316.

Zhou, L. *et al.* (2012) ‘A parallel genome-wide mRNA and microRNA profiling of the frontal cortex of HIV patients with and without HIV-associated dementia shows the role of axon guidance and downstream pathways in HIV-mediated neurodegeneration’, *BMC Genomics*, 13, p. 677. Available at: https://doi.org/10.1186/1471-2164-13-677.
