# Supplementary material for: New genetic insights into HIV-associated neurocognitive disorder and Alzheimer's disease
Source: Genes Dis. 2025 Feb 26;12(5):101576. doi: 10.1016/j.gendis.2025.101576 (PMC12142519; doi:10.1016/j.gendis.2025.101576)
Supplement: Multimedia component 5 [file mmc5.docx]

**Table S12. Summary of Key Findings from Differential Gene Expression Analysis in HIV-Associated Neuroinflammation and HAND: Gene Identification, Pathways, and Validation Across Studies.**

| **Indicators** | **Figure/Table Reference** | **Key findings/description** |
| --- | --- | --- |
| Workflow for methods | Figure S1 | Detailed workflow for identifying and analyzing key genetic insights into HAND and Alzheimer's Disease |
| Differentially Expressed Genes (DEGs) in HIV-Infected Brain Tissue | Figure S2A | **16 DEGs** identified across four or more studies, consistently altered in HIV-infected brain tissues. These include: |
|  |  | 14 (88%)—B2M, HLA-C, IFI6, and MX1—exhibited consistent directional changes across all comparisons and brain regions  - HLA-C (Human Leukocyte Antigen)  - B2M (Major Histocompatibility Complex Class I)  - IFI6 (Interferon Induced Protein 6)  - MX1 (MX Dynamin Like GTPase 1) |
|  |  | - **Decreased expression**: **SYN2** (Synapsin II)  - while DTNA, IFI44, IFIT1, IFIT3, IFITM1, ISG15, and LGALS3BP were the most common increased |
|  | Figure S2B | **438** biomarkers were identified in HAND versus HIV, 133 in HIVE versus HIV, and 1,861 in MCD versus cognitively healthy HIV individuals. |
|  |  | Three shared biomarkers (**STAT1, IFI35**, and **CDC2L2**) were found between HAND versus HIVE versus HIV alone. **STAT1** and **IFI35** exhibited inconsistent directional changes related to HIV infection across different studies |
| HAND and healthy controls | Figure S3A | - Genes involved in innate immune response and cytokine signaling were upregulated in HAND compared to healthy controls.  - Interferon response, Cytokine signaling, Synaptic function and axon integrity pathways were downregulated. |
| HAND with ART and healthy controls | Figure S3B | - Increased expression of proteins involved in viral response and interferon signaling in HAND patients on ART.  - a decrease in proteins associated with chromatin organization, ribonucleoprotein complex biogenesis, nuclear export, and translation |
| HAND+HIVE and healthy controls | Figure S3C | - The pathogenesis of the disease is associated with increased response to viral proteins and interferon signaling proteins, indicating activation of defense mechanisms following HIV's invasion of the CNS.  - There was also a notable reduction in synaptic, axonal, and neuronal projection proteins in the brains of individuals with HAND+HIVE |
| HAND and HIV | Figure S3D | - Compared to individuals with HIV alone, those with HAND exhibited increased levels of proteins associated with memory impairment and decreased levels of proteins related to glutamine metabolism, retrograde transport at the trans-Golgi network, and functions of axons and synapses |
| HAND+HIVE and HIV | Figure S3E | - In individuals with HAND+HIVE, there was significant evidence of increased levels of proteins associated with interferon signaling; however, these proteins did not regulate viral genome replication.  - There was a notable decrease in proteins related to axonal function and synaptic integrity in HAND+HIVE individuals compared to individuals with HIV but no cognitive impairment |
| MCD vs. CNHIV | Figure S3F | - Compared to NCHIV individuals, those with MCD exhibited higher levels of proteins associated with membrane trafficking and axon guidance, but lower levels of proteins related to nucleolus function and hypothalamic systems |
| Association between HIV infection and Alzheimer’s disease (AD) | Figure S4A-B | - There were 262 shared genes implicated in the pathogenesis of AD related to HIV infection, proteins that were also found to be associated with synapse activity |
|  | Figure S4C-E | - Proteins associated with specific signaling pathways were significantly enriched in HAND, including synaptic transmission, dopaminergic signaling, catecholamine signaling, and neuron projection, many of which are known to be involved in AD |
|  | Figure S4F-G | - Identified **7 key proteins** (**APP**, **MAPT**, **MAPK3**, **AKT1**, **APOE**, **JUN**, **SNCA**) with central roles in AD and HIV, involved in **synaptic function**, **memory impairment**, and **neurodegeneration**.  - These proteins were found to be increased in both HIV and AD, linked to **amyloid plaques** and **neurofibrillary tangles** in the brain.  - Expression levels of 5 proteins (**APP**, **MAPT**, **MAPK3**, **APOE**, **SNCA**) in HIV-infected brains and their association with AD neuropathological features such as amyloid plaques and neurofibrillary tangles.  - These proteins were validated using NeuPro database as well as manual research on Pubmed. |
| Analysis of important proteins implicated in AD pathogenesis due to HIV | Figure S5 | - Important proteins identified using three centrality indicators related to synaptic proteins, mitochondrial proteins, and proteins located at synapses.  Ex: SNAP25, COX5A, CST3, APOE, GLRX, APP, CLU, SOD2, etc. |
| Cross-referencing data from multiple databases for AD-related proteins (HAND, NeuroPro, GeneCard, and MalaCards) | Figure S6A-E | - This analysis revealed 95 shared genes, with eight important proteins—APOE, SNCA, DLG4, GSK3B, APP, SNAP25, GAPDH, and GFAP—highlighted using three centrality metrics.  - Significant associations of these proteins with various biological processes, cellular components, molecular functions, and diseases, many of which are linked to AD.  - Protein-protein interaction enrichment (PPIE) analysis identified key pathways, such as those related to AD, neurodegeneration, and synaptic transmission, as critical processes potentially connecting AD to HIV infection  - Proteins implicated in the pathogenesis of AD caused by HIV are shown in Figure 5E. |
| Validation of HAND-related genes using single-cell RNA sequencing (scRNA-seq) | Figure 7A-D | Validation of **95 HAND-related genes** in recent scRNA-seq datasets (GSE233717 and GSE202410), showing **increased expression** of key genes: |
|  |  | - **APOE**, **APP**, **GAPDH**, **HSPA8**, **CTSB** in **immune cells** (e.g., CD4 T cells, B cells, CD14 monocytes). These genes were highly expressed across different cell types, confirming their association with HAND pathology. |
| Validation of HAND-related genes in CSF samples using scRNA-seq | Figure S8A-D | Validation in **CSF samples** from HIV-infected individuals showed **52 shared DEGs** with the 95 HAND-related genes, including **APP**, **GSK3B**, **RHOA**, **GAPDH**, and **SNCA**. These genes exhibited significant expression across immune cell populations, particularly in **CD4 T cells**, **B cells**, and **monocytes**. |
| Comparison of DEGs between HIV-infected and healthy CSF samples | Figure S9A-B | - The healthy control group showed 6,003 DEGs, 44 of which overlapped with the 95 HAND-related DEGs.  - Centrality indicators identified 6 key DEGs, among which SOD1 and GAPDH exhibited high expression across all immune cell populations in the CSF, while APOE and SNCA were mostly expressed in B cells.  - The DEGs in healthy controls were found in memory CD4 T cells, CD8 T cells, CD14 monocytes, and NK cells |
| Regulatory Network of DEGs, miRNAs, and Transcription Factors (TFs) | Figure S10A-E | **miRNA-TF-DEG network analysis** showed key regulatory interactions: |
|  |  | - **39 DEGs** were linked to **miRNAs** (e.g., **hsa-miR-16-5p**, **hsa-miR-320a**, **hsa-miR-335-5p**) |
|  |  | - **Key TFs**: **THRA**, **NEUROD6** were central to the network, regulating **neuroinflammation**, **synaptic signaling**, and **AD-related pathways**. |
| Genetic Variants (SNPs) Associated with HAND | Figure S11A-B | **80 SNPs** associated with HAND were identified from six databases. Key SNPs: |
|  |  | - **MTND4P3 (rs4718789-T)**, **MSH6 (rs2098242-T)**, **RNA5SP231 (rs4718789-T)**. These SNPs were linked to **immune response**, **neurodegeneration**, and **synaptic function** pathways, highlighting their potential role in the pathogenesis of HAND. |
| HAND database | Table S1 | **Table S1.** Studies included in HAND database |
| Biomarkers changes involved in the pathogenesis of AD caused by HIV | Table S2A-G | - Supplementary Data 2. HAND Database (Biomarkers changes involved in the pathogenesis of AD caused by HIV)  - Supplementary Data 2A. HIV+ HAND vs. Uninfected controls  - Supplementary Data 2B. HIV+ HAND_ART vs. Uninfected  - Supplementary Data 2C. HIV+ HAND HIVE+ vs. Uninfected  - Supplementary Data 2D. HIV+ vs. HIV+ HAND  - Supplementary Data 2E. HIV+ HIVE vs. HIV+ no HIVE  - Supplementary Data 2F. HIV+ vs. Uninfected controls  - Supplementary Data 2G. HIV+ MND vs. HIV+with cognitive normal |
| Biomarkers associated with Alzheimer's disease | Table S3 | Supplementary Data 3. Biomarkers associated with Alzheimer's disease |
| Biomarkers associated with HIV | Table S4 | Supplementary Data 4. Biomarkers associated with HIV |
| Overlapped biomarkers associated with HIV and Alzheimer's disease | Table S5 | Supplementary Data 5. Overlapped biomarkers associated with HIV and Alzheimer's disease |
| Overlapped biomarkers (genes related to HIV and dementia) and NeuroPro database involved in the pathogenesis of Alzheimer's disease | Table S6 | Supplementary Data 6. The association between overlapped biomarkers (genes related to HIV and dementia) and NeuroPro database involved in the pathogenesis of Alzheimer's disease |
| Overlapped biomarkers (genes related to HIV and Azlheimer's disease) and NeuroPro database involved in different clinical stages of AD | Table S7 | Supplementary Data 7. The association between overlapped biomarkers (genes related to HIV and Azlheimer's disease) and NeuroPro database involved in different clinical stages of AD |
| Key overlapped biomarkers changed in HIV individual's brain | Table S8 | Supplementary Data 8. Key overlapped biomarkers changed in HIV individual's brain |
| Validation of miRNAs experimentally to target 95 HAND-associated genes | Table S9 | Supplementary Data 9. Validation of miRNAs experimentally to target 95 HAND-associated genes using BRAINSPAN dataset (https://www.brainspan.org/static/download.html) |
| Validation of miRNAs experimentally | Table S10 | Supplementary Data 9. Validation of miRNAs experimentally to target 95 HAND-associated genes using miRTarBase |
| Genome-wide association studies | Table S11 | Supplementary Data 10. Combined human genome-wide association studies implicated in the pathogenesis of Alzheimer's disease caused by HIV |
| Summary of Key Findings | Table S12 | Summary of Key Findings from Differential Gene Expression Analysis in HIV-Associated Neuroinflammation and HAND: Gene Identification, Pathways, and Validation Across Studies |
