# Supplementary figures and images for: New genetic insights into HIV-associated neurocognitive disorder and Alzheimer's disease
Source: Genes Dis. 2025 Feb 26;12(5):101576. doi: 10.1016/j.gendis.2025.101576 (PMC12142519; doi:10.1016/j.gendis.2025.101576)

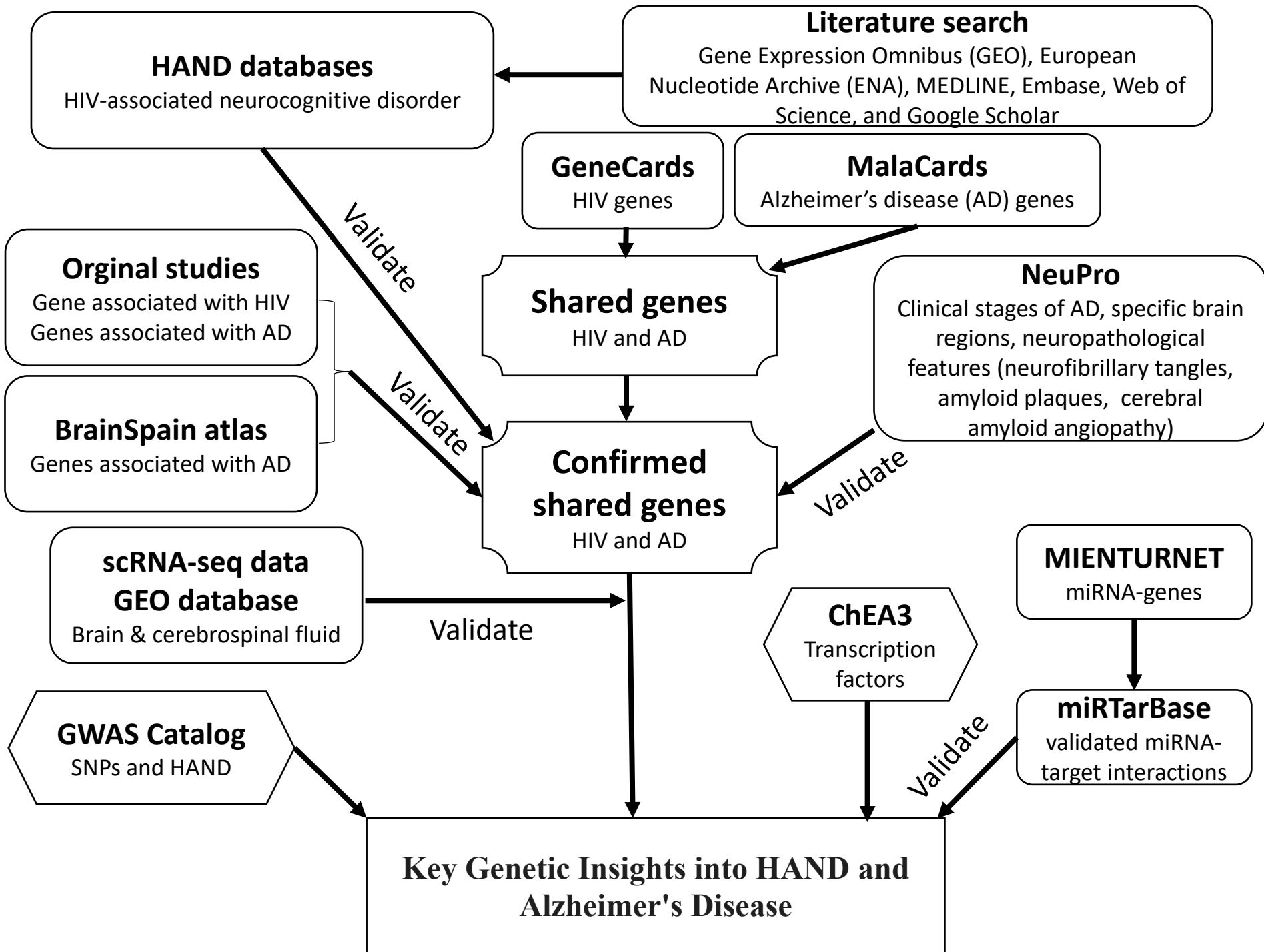

Supplement: Multimedia component 6 [file mmc6.pdf]

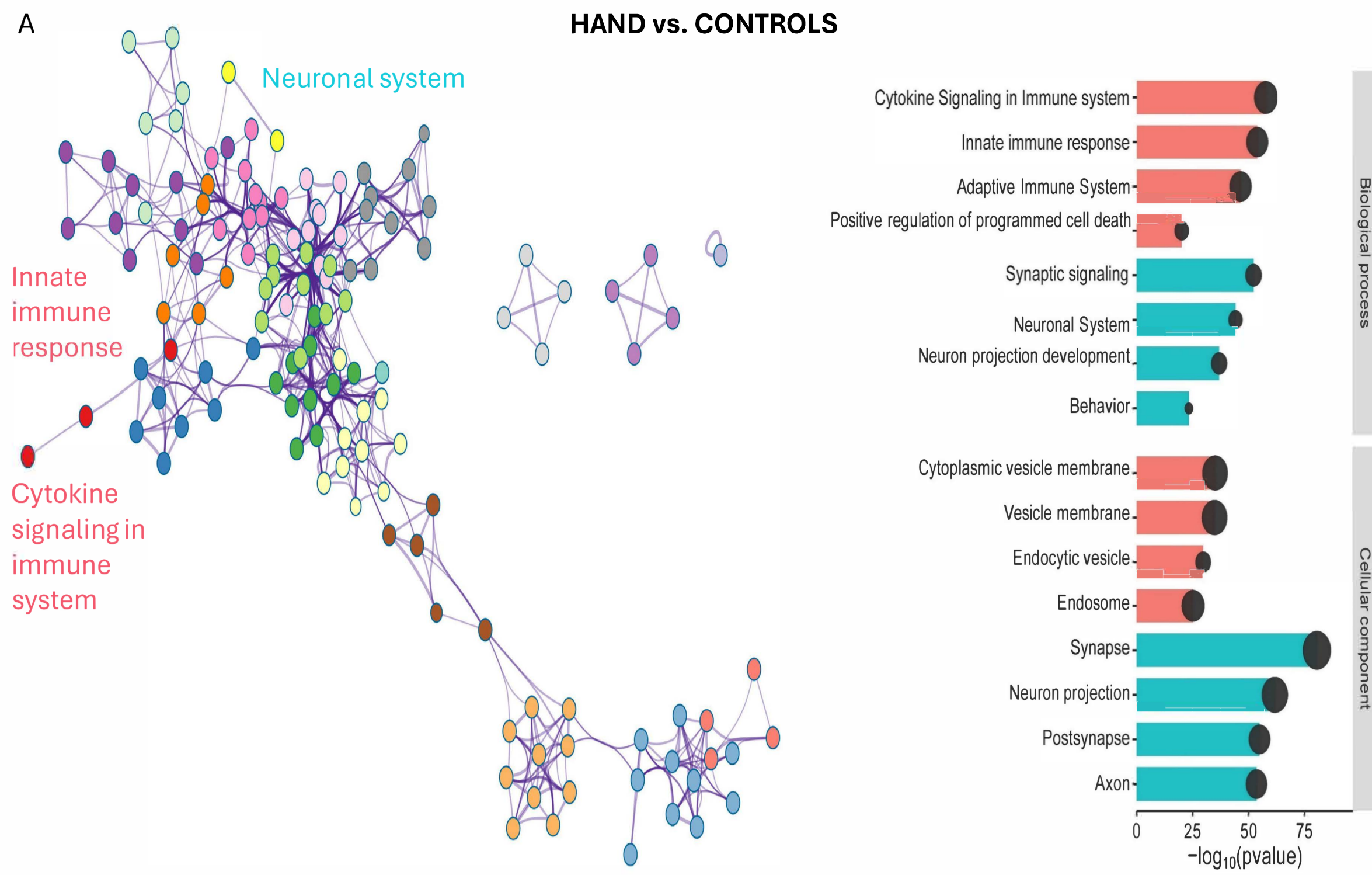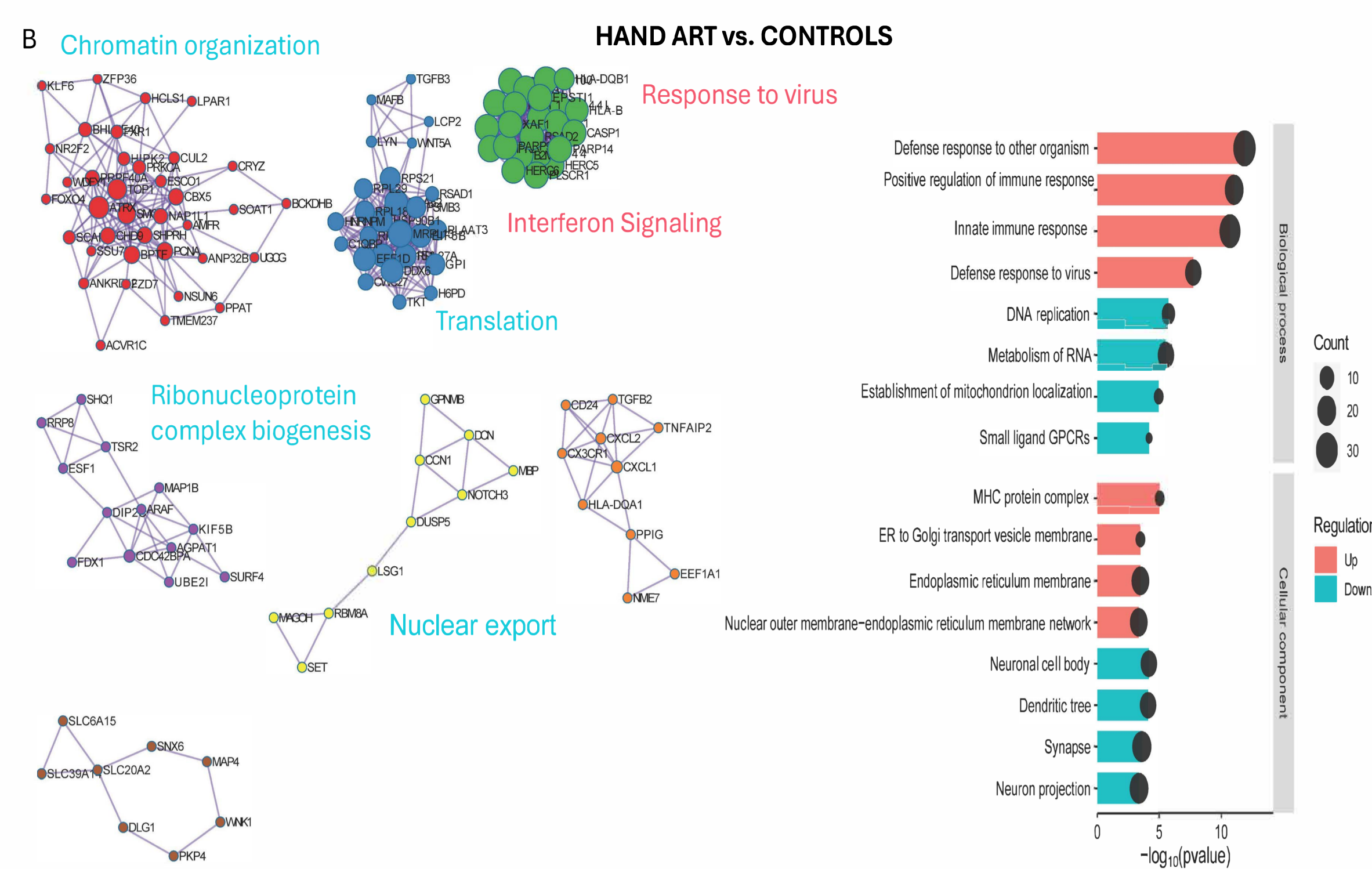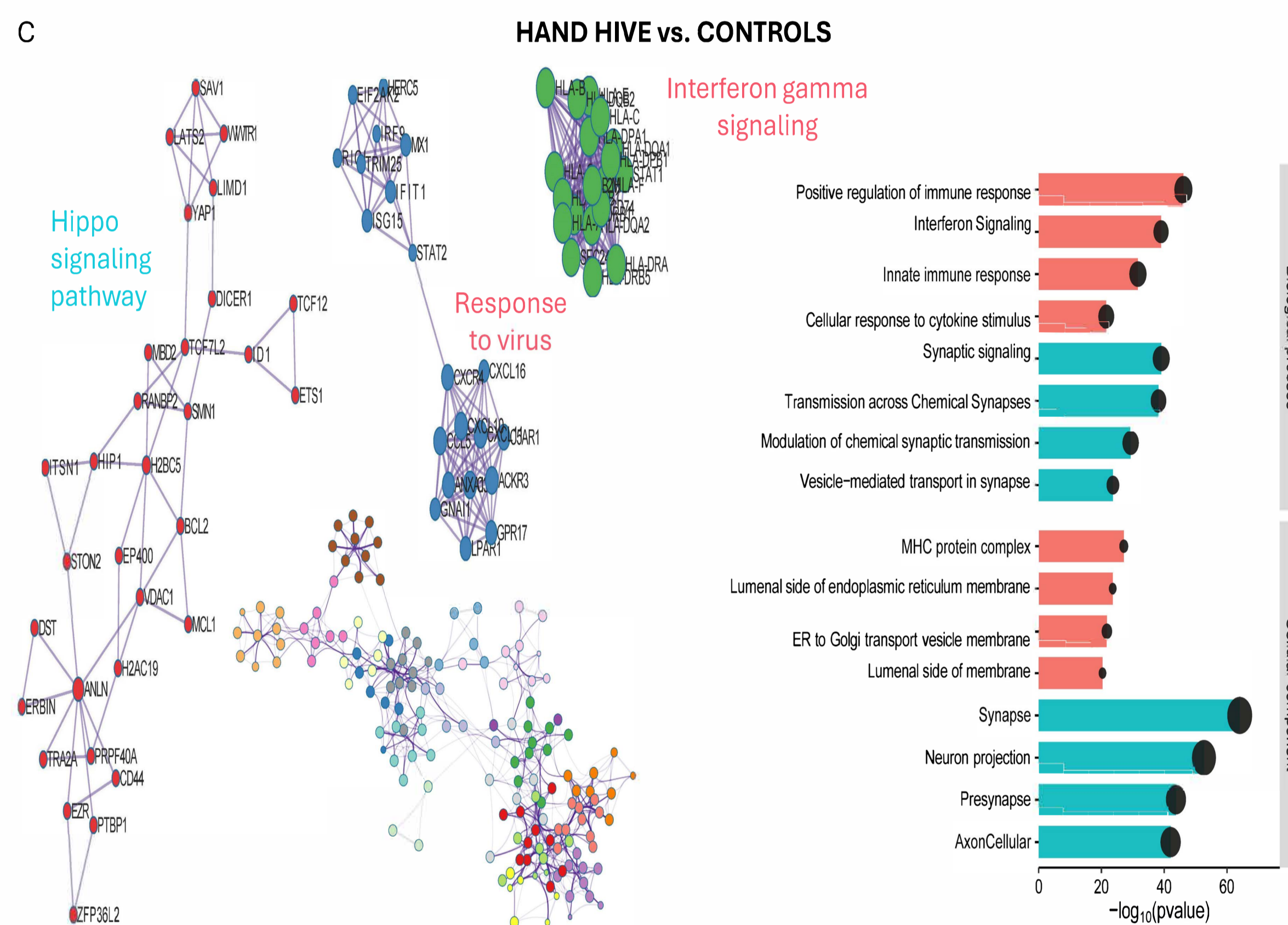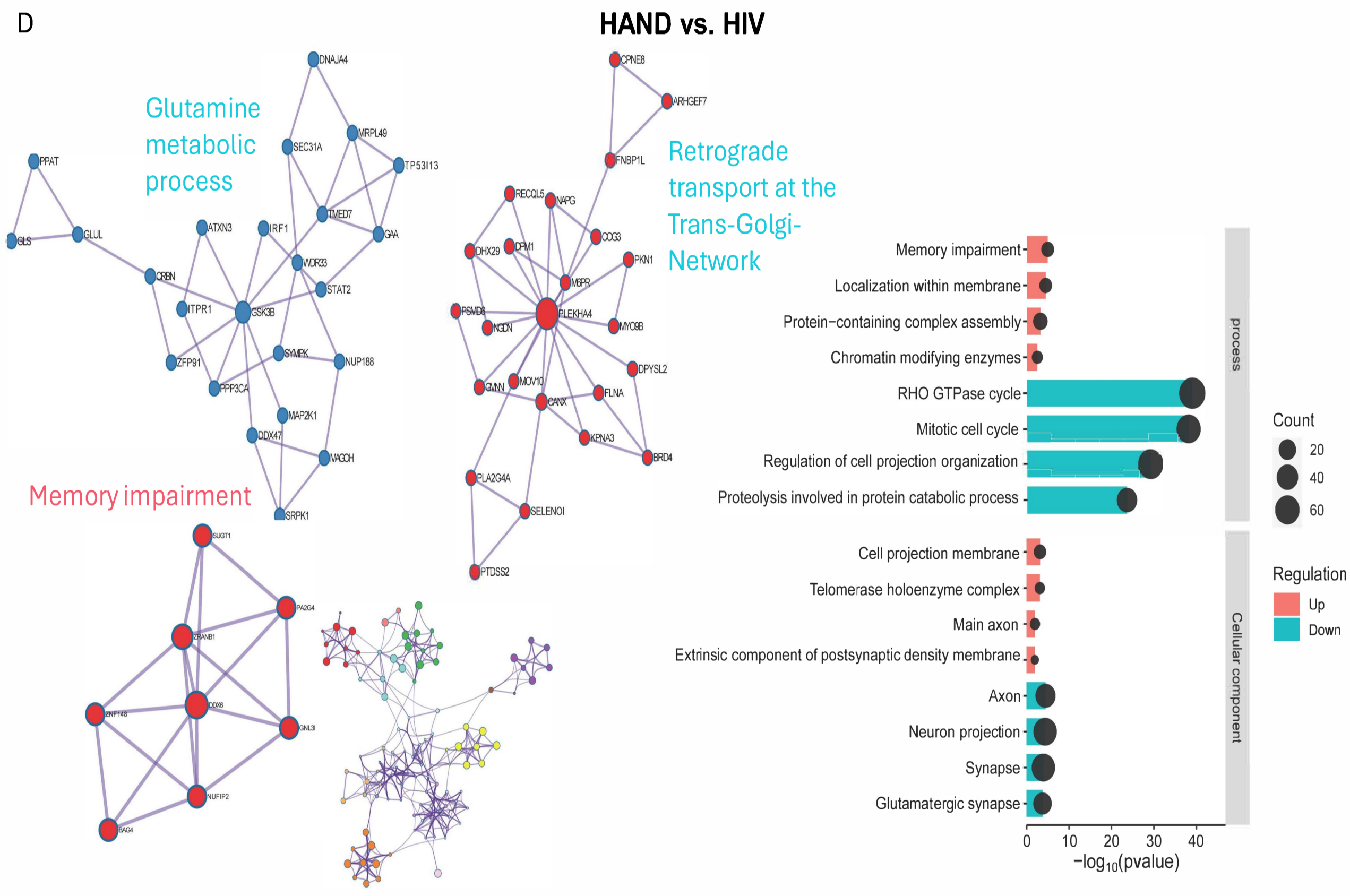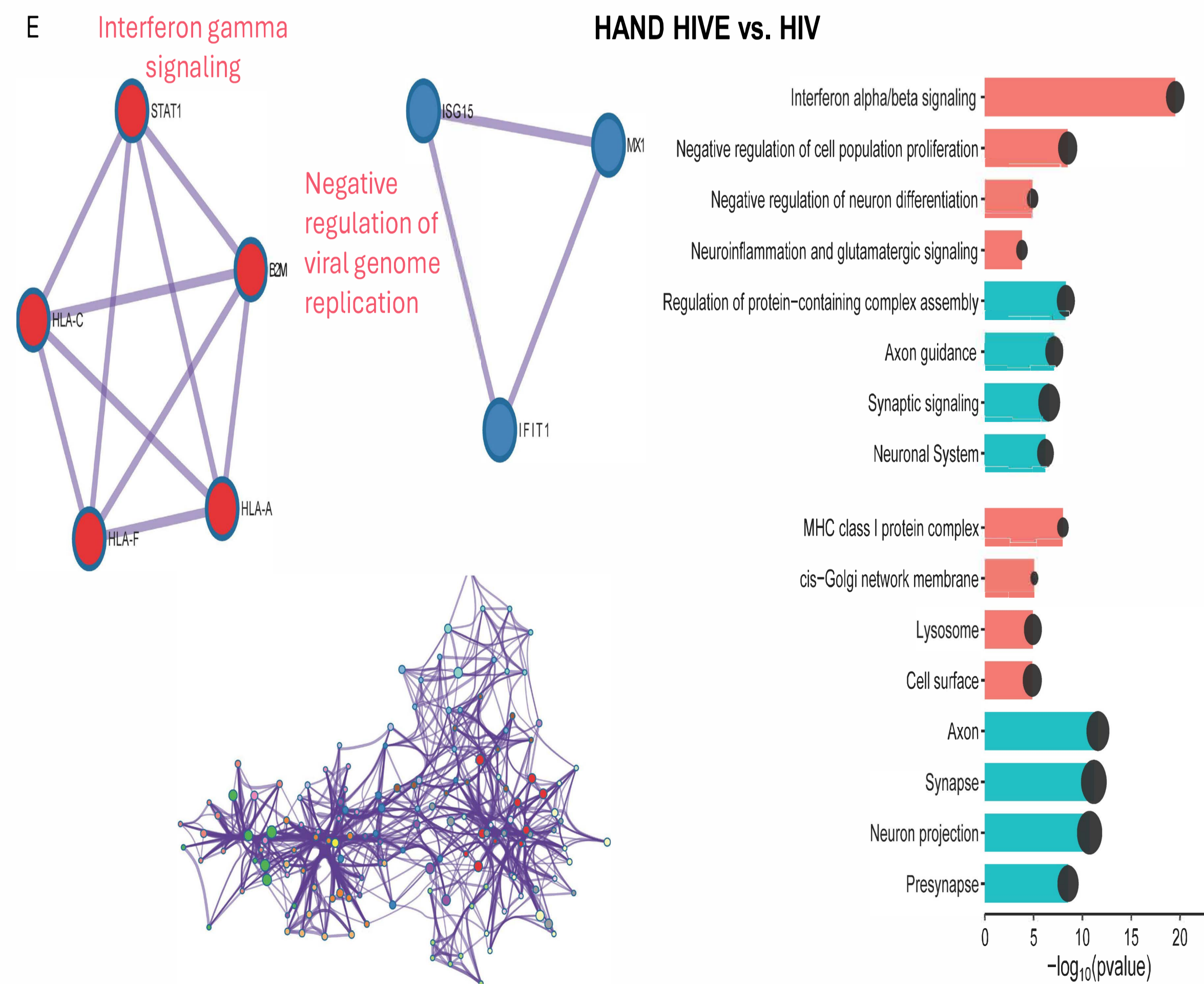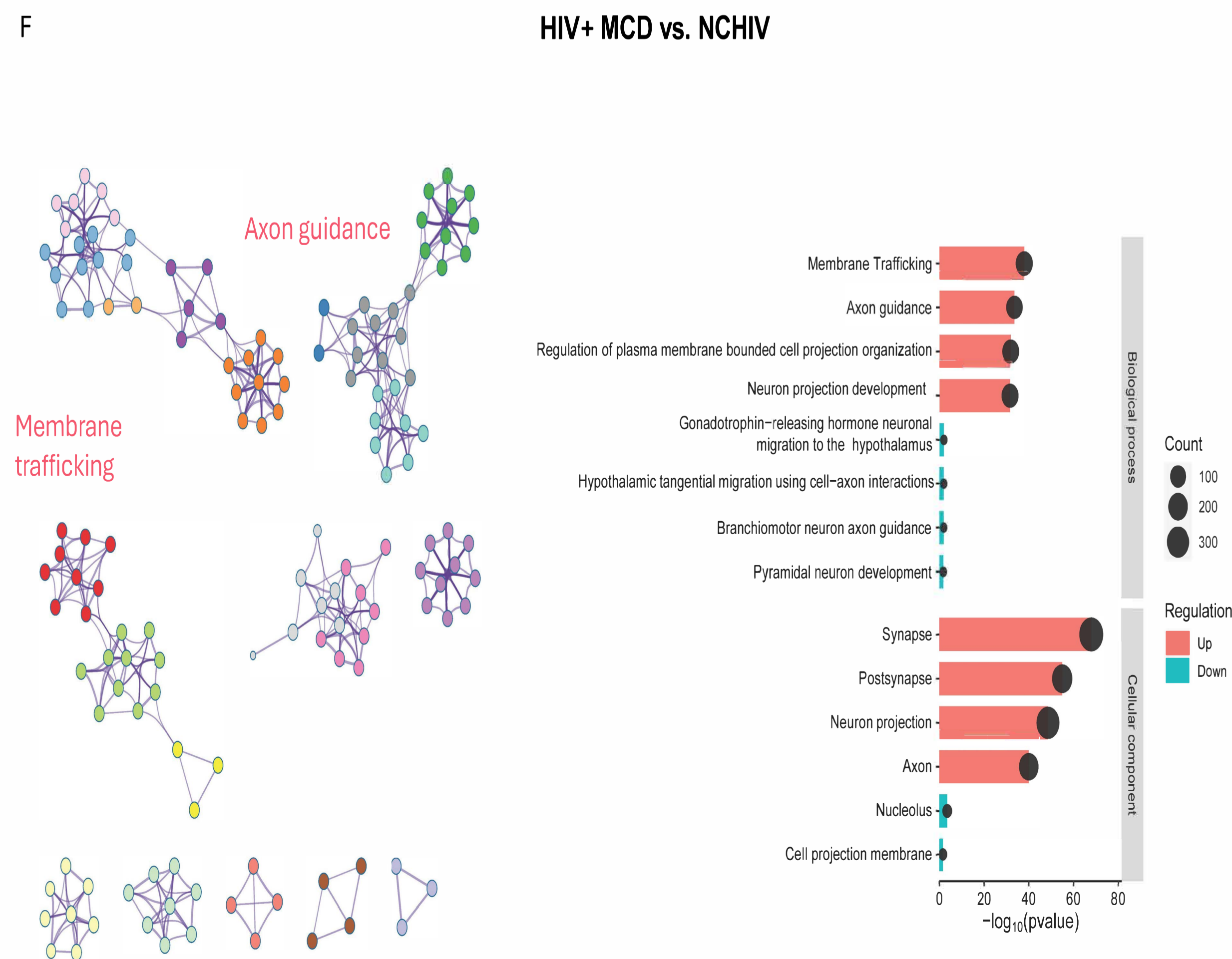

Supplement: Multimedia component 8 [file mmc8.pdf]

A

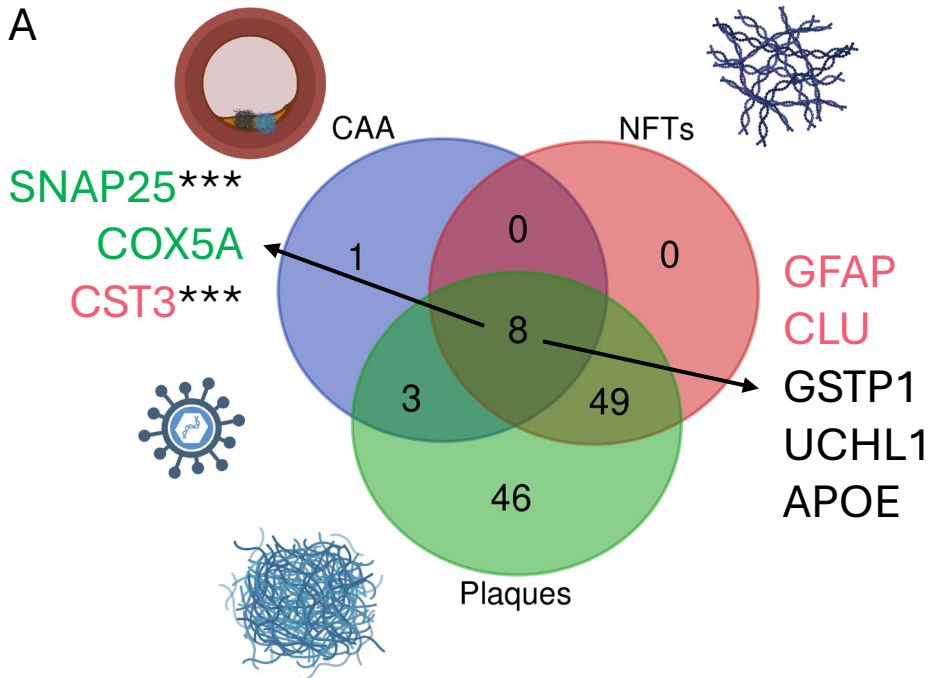

B

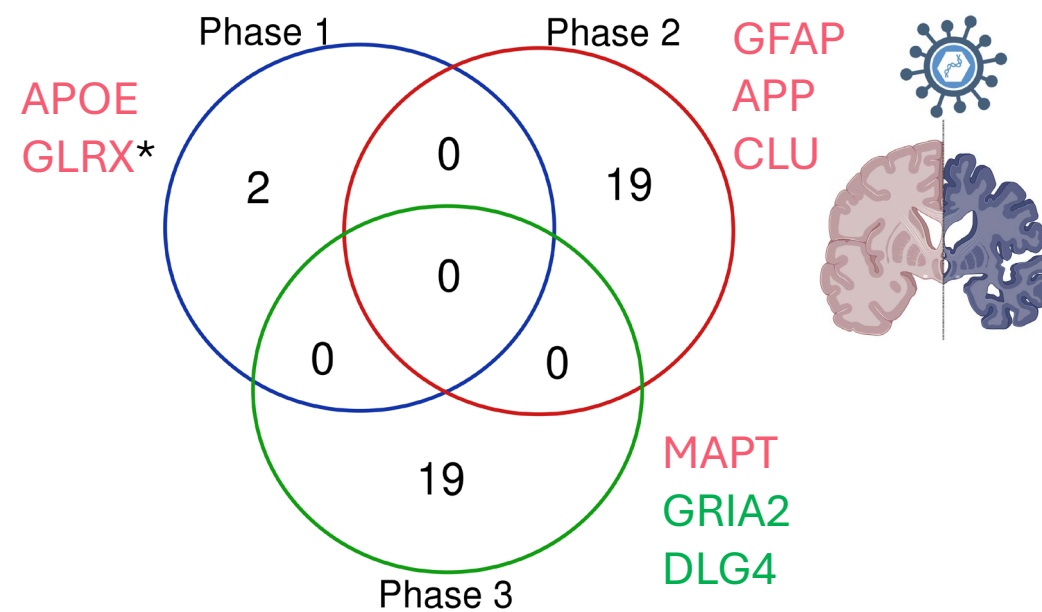

C

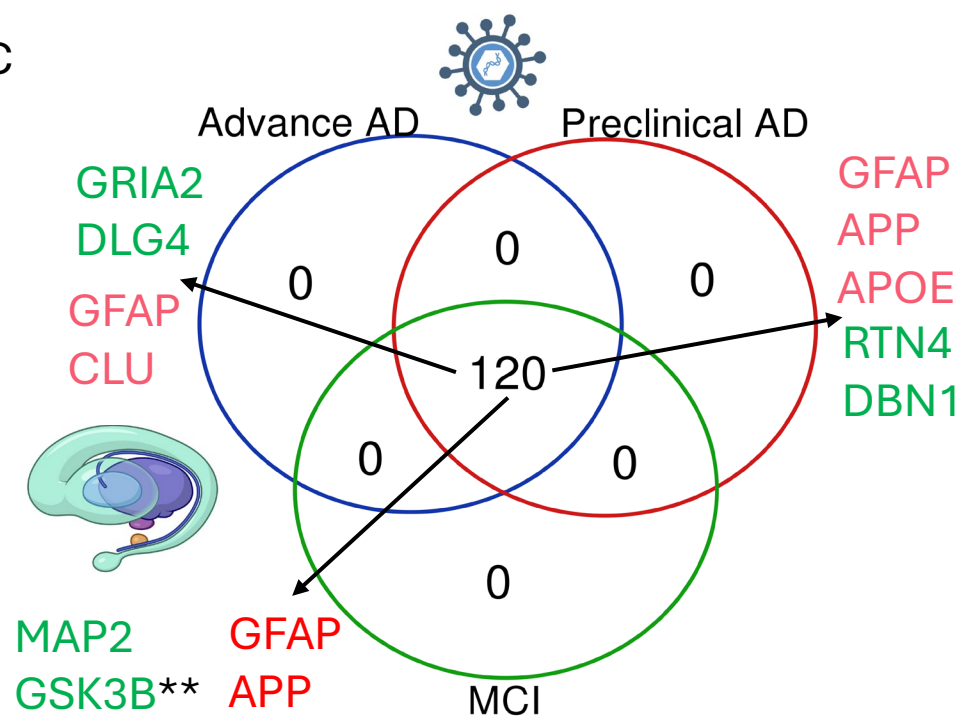

D

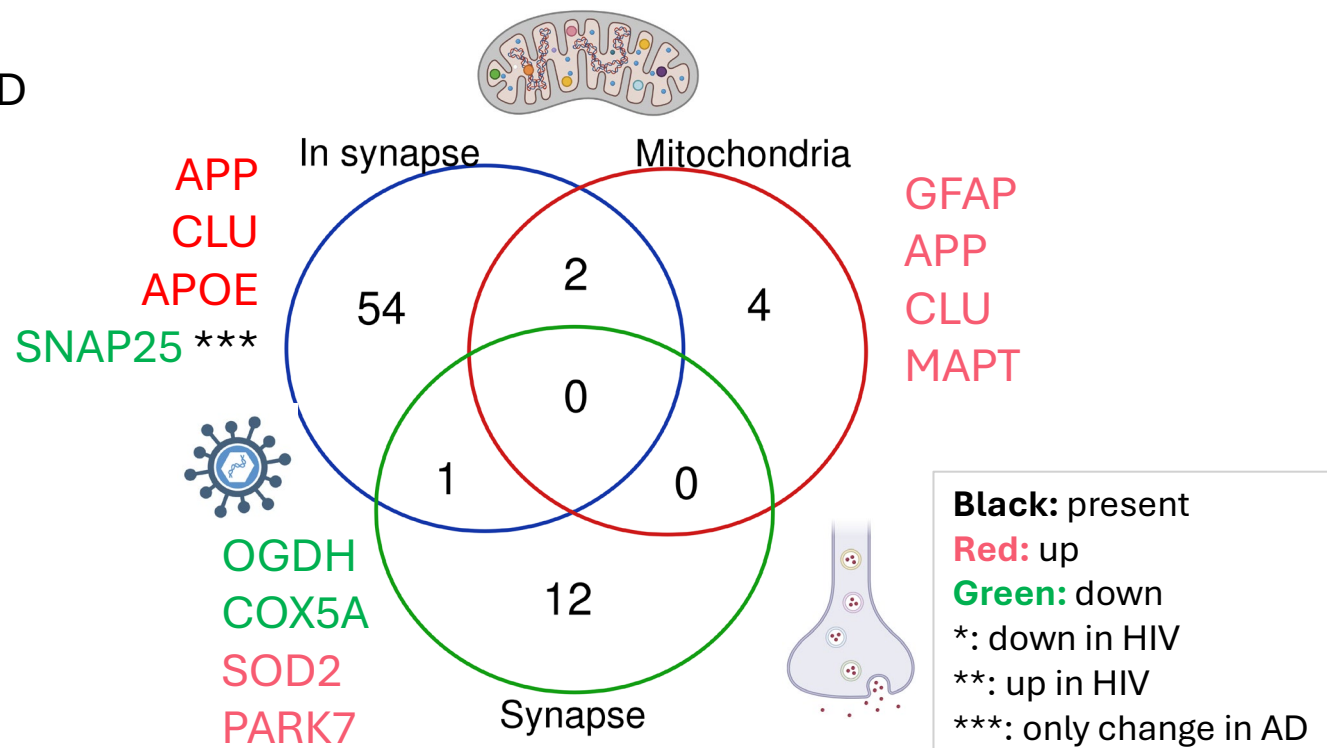

Supplement: Multimedia component 10 [file mmc10.pdf]

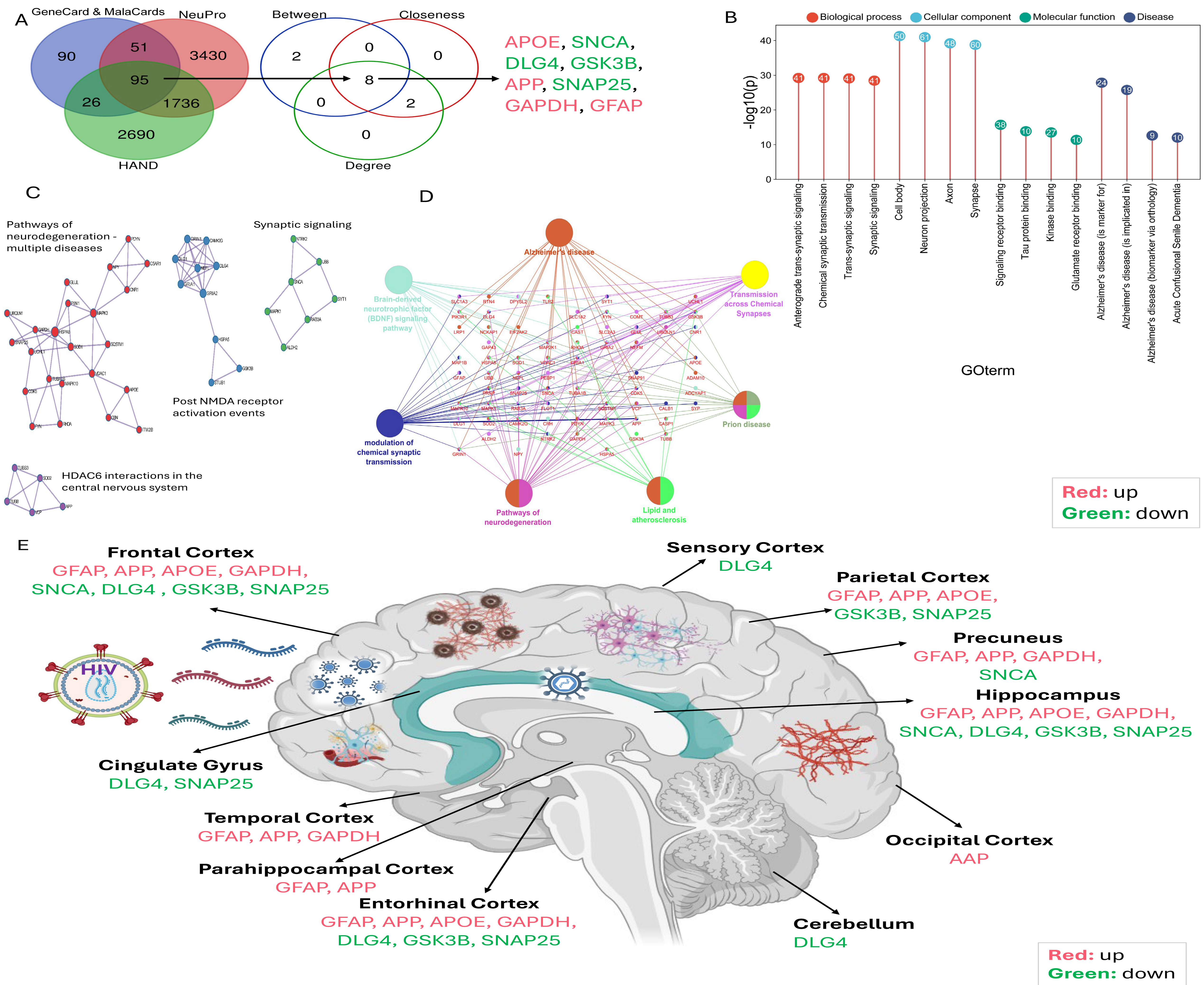

Supplement: Multimedia component 11 [file mmc11.pdf]

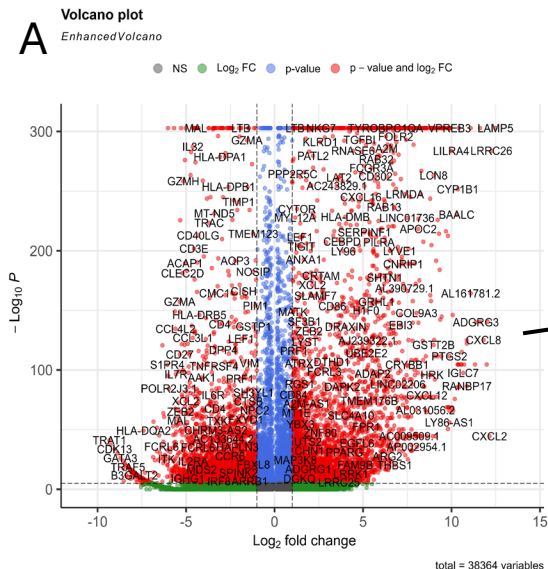

## CSF in HIV individuals

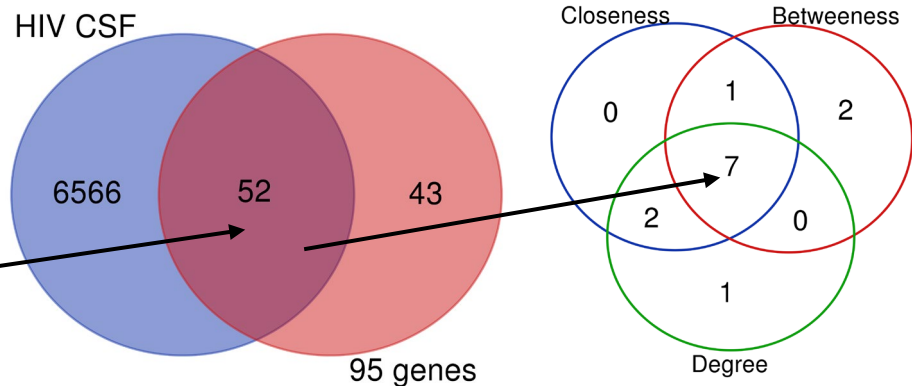

**B**

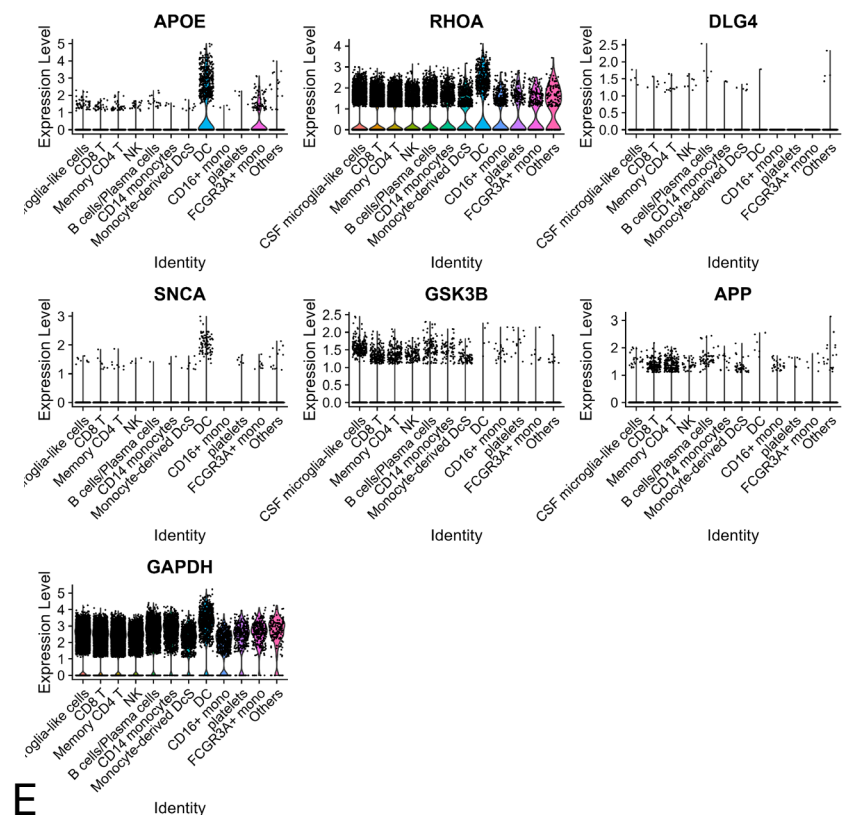

**C**

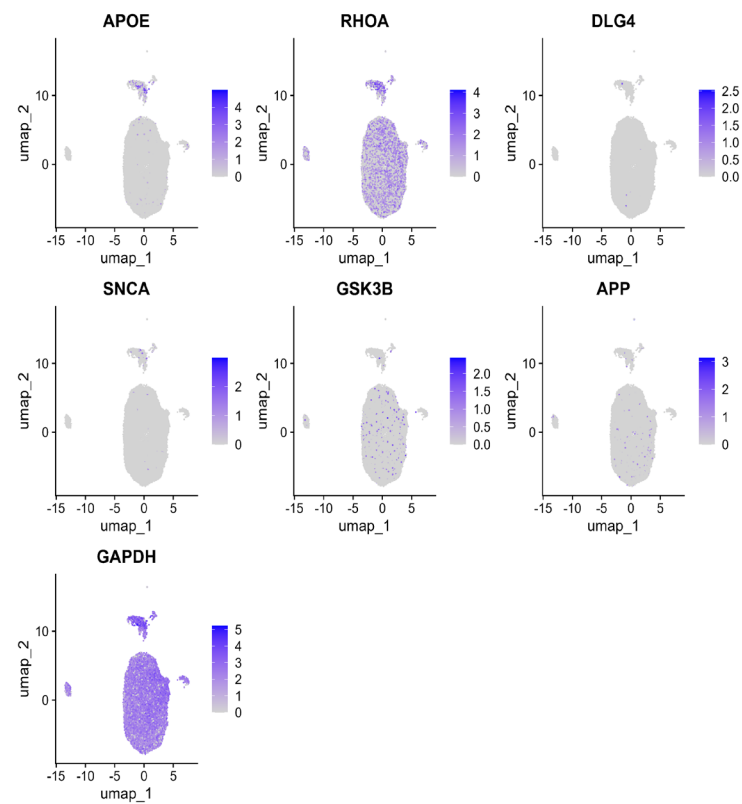

**D**

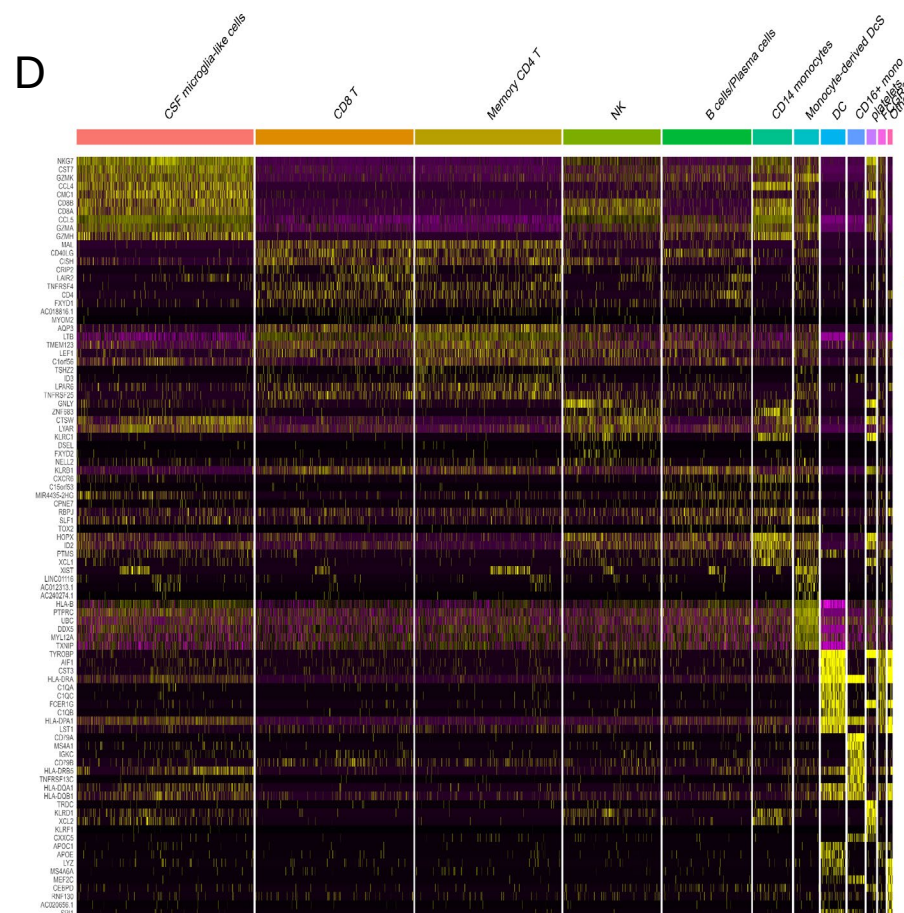

**E**

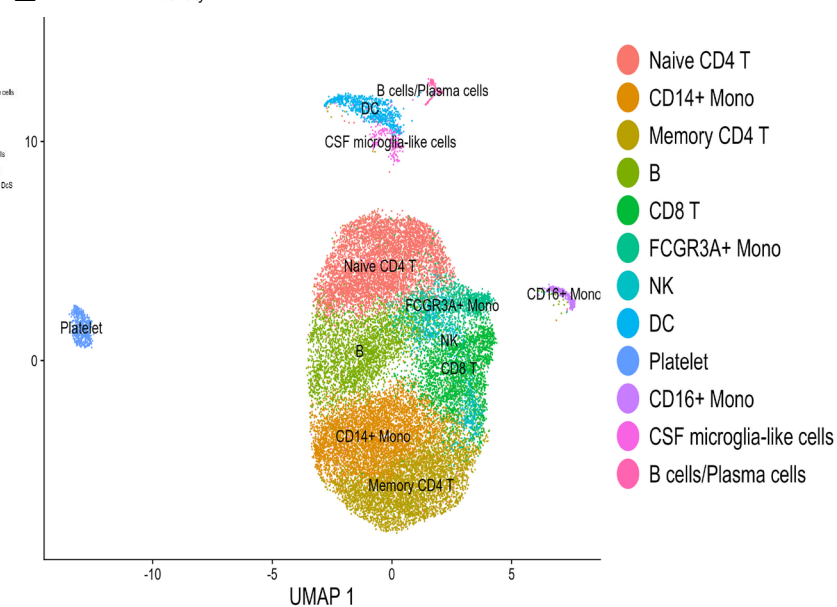

Supplement: Multimedia component 13 [file mmc13.pdf]

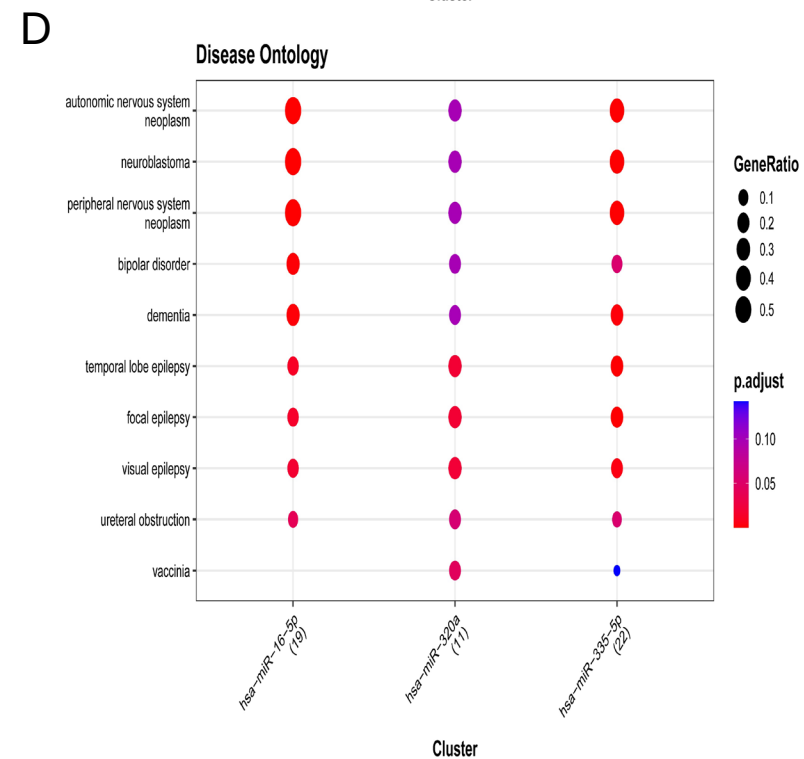

Supplement: Multimedia component 15 [file mmc15.pdf]
