# Supplementary material for: New genetic insights into HIV-associated neurocognitive disorder and Alzheimer's disease
Source: Genes Dis. 2025 Feb 26;12(5):101576. doi: 10.1016/j.gendis.2025.101576 (PMC12142519; doi:10.1016/j.gendis.2025.101576)

A

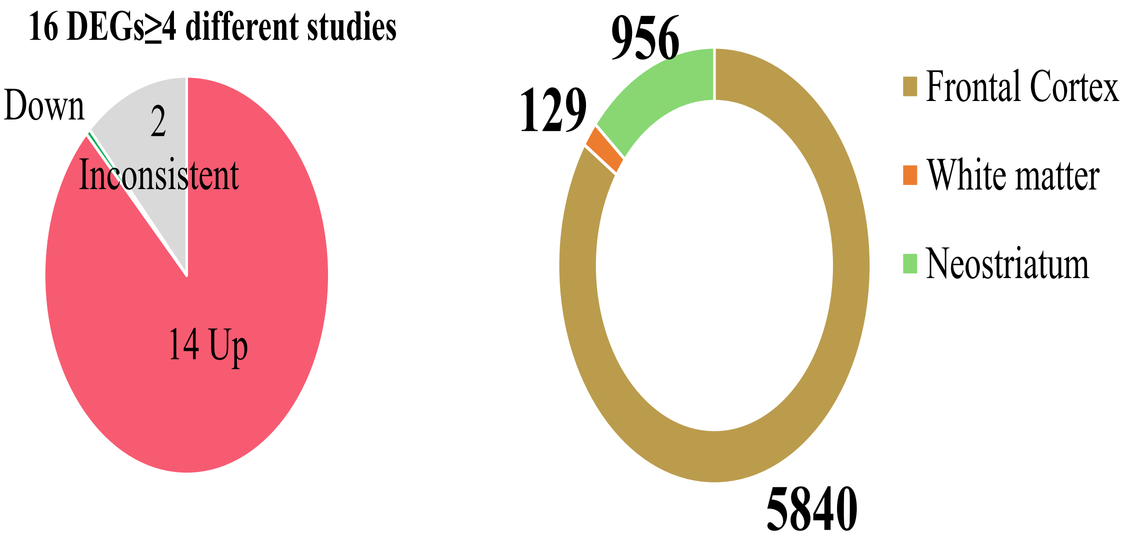

	Score	Frontal Cortex	Neostriatum	White matter	HIV+ HAND HIVE vs. controls	HIV+ HAND vs. controls	HIV+ HAND_ART vs. controls	HIV+ HIVE vs. HIV+ no HIVE	HIV+ vs. controls	HIV+ vs. HIV+ HAND	HIV+ MND vs. CNHIV+
ATP6V1A	4	+	+		↓			↓			↑
B2M	5	+	+	+	↑	↑	↑	↑			↑
BST2	6	+	+		↓	↓		↑			↑
DTNA	4	+	+		↑↑↑↓	↑		↑	↑		
GLRB	4	+		+	↓	↑				↓	
HLA-C	4	+	+	+	↑	↑		↑			
IFI44	4	+	+	+	↑↑↑↓	↓	↑	↑			
IFI6	4	+	+		↑	↑					↑
IFIT1	4	+	+		↑↓	↑↓	↑	↑			
IFIT3	4	+	+	+	↑↑↑↓	↑↑↓	↑	↑			
IFITM1	5	+	+		↑↑↑↓	↑↑↑↓		↑	↑		
ISG15	5	+	+	+	↑↑↑↓	↑↓		↑	↑		
LGALS3BP	5	+	+		↑↑↑↓	↑↓		↑			↑
MX1	4	+	+	+	↑	↑	↑	↑			
STAT1	4	+	+	+	↑	↑	↑	↑		↓	
SYN2	4	+			↑↓	↓		↓			↑

Back: present
Red: up
Green: down

B

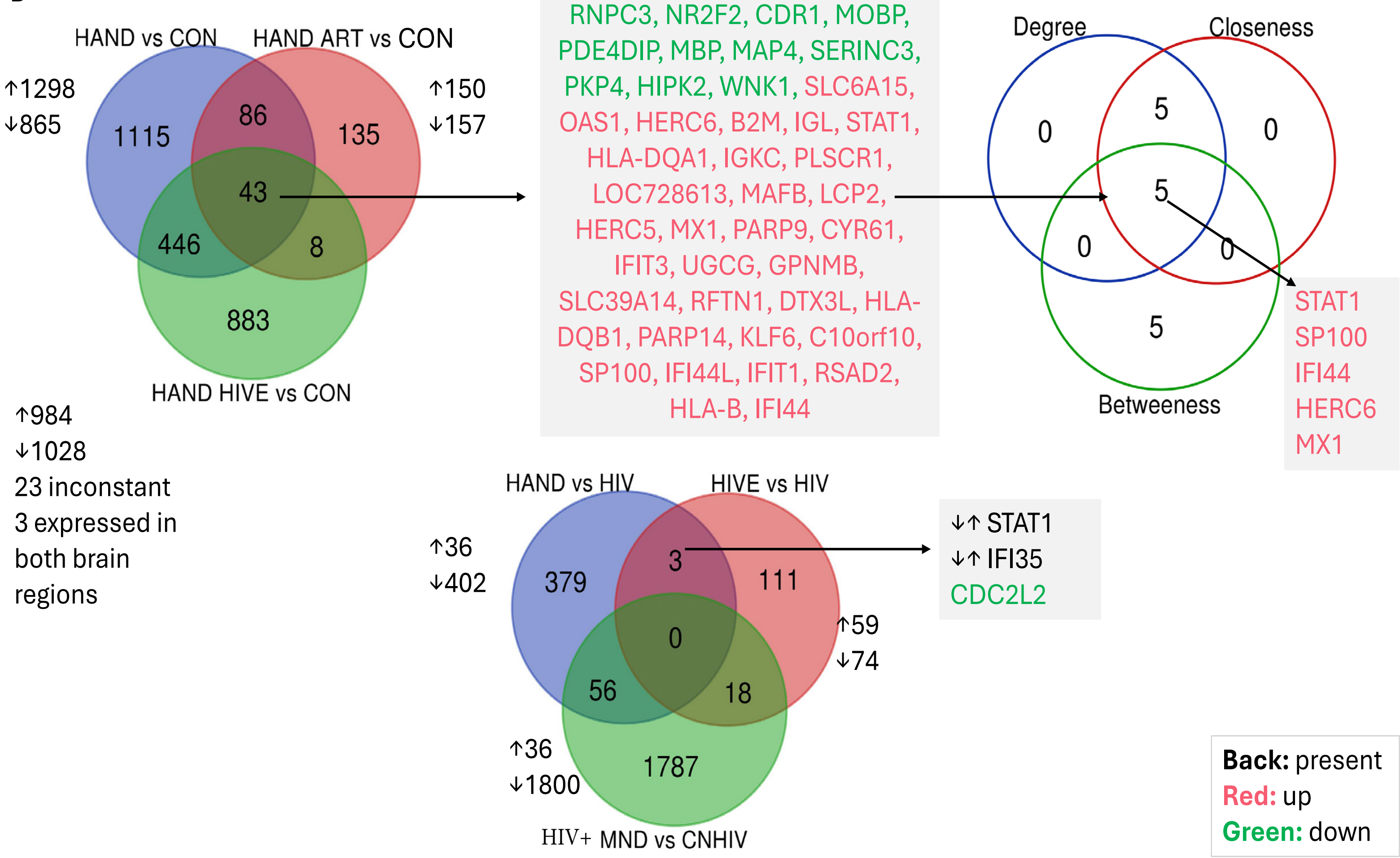

Supplement: Multimedia component 7 [file mmc7.pdf]
