# Supplementary material for: New genetic insights into HIV-associated neurocognitive disorder and Alzheimer's disease
Source: Genes Dis. 2025 Feb 26;12(5):101576. doi: 10.1016/j.gendis.2025.101576 (PMC12142519; doi:10.1016/j.gendis.2025.101576)
Supplement: Multimedia component 9 [file mmc9.pdf]

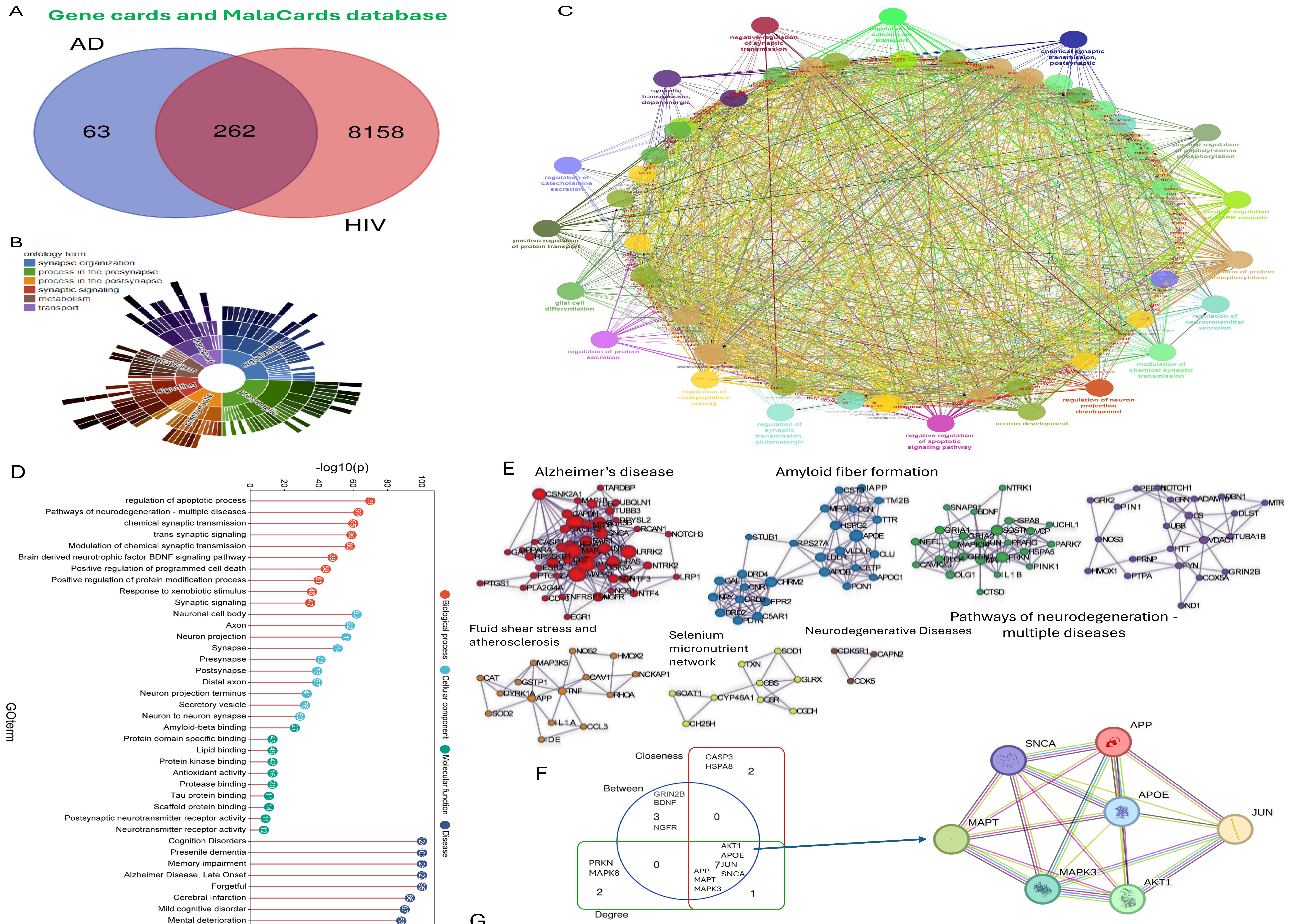

G

	Alzheimer's Disease								HIV	
Key biomarkers	NeuroPro Score (Total)	Changes in AD	NFT Protein	Plaque Protein	CAA Protein	Published Studies Linked to AD	Disease Phase	Changes in HIV	Published Studies Linked to HIV individual's brain	
APP	28	Increase	Increase	Increase		18228	Phase 2: Early AD	Increase	6	
APOE	19	Increase	Present	Increase	Increase	10606	Phase 1: Pre-neuropathology	Increase	10	
MAPT	16	Increase	Increase	Increase		23171	Phase 3: AD	Increase	1	
SNCA	7		Present	Present				Increase	1	
MAPK3	7			Present	Increase			Increase	2	
