# Supplementary material for: New genetic insights into HIV-associated neurocognitive disorder and Alzheimer's disease
Source: Genes Dis. 2025 Feb 26;12(5):101576. doi: 10.1016/j.gendis.2025.101576 (PMC12142519; doi:10.1016/j.gendis.2025.101576)

A

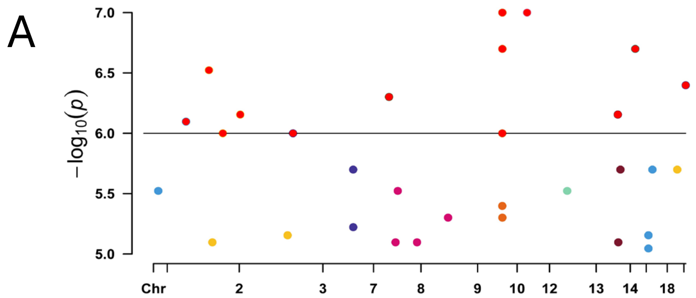

B

MAPPED GENE	STRONGEST SNP-RISK ALLELE	P-VALUE	OR or BETA	95% CI	DISEASE/TRAIT
**MTND4P3**	**rs4718789-T**	**0.0000005**	**4.44**	**4.67-4.21**	HAND (mild neurocognitive disorder)
**RNA5SP231**	**rs4718789-T**	**0.0000005**	**4.44**	**4.67-4.21**	HAND (mild neurocognitive disorder)
**MSH6**	**rs2098242-T**	**0.000008**	**3.18**	**3.41-2.95**	HAND (mild neurocognitive disorder)
SPAG16	rs35824328-C	0.000007	2.79	3.02-2.56	HAND (mild neurocognitive disorder)
SPAG16	rs35824328-C	0.000007	2.79	3.02-2.56	HAND (mild neurocognitive disorder)
IL4R	rs1859308-G	0.000002	2.49	2.72-2.26	HAND (asymptomatic neurocogntive impairment)
IL21R	rs1859308-G	0.000002	2.49	2.72-2.26	HAND (asymptomatic neurocogntive impairment)
Y_RNA	rs7863010-C	0.000001	2.25	2.48-2.02	HAND
PTPN3	rs7863010-C	0.000001	2.25	2.48-2.02	HAND
CSMD1	rs7840128-A	0.000008	2.17	2.40-1.94	Neurocognitive impairment in HIV-1 infection (dichotomous)

C

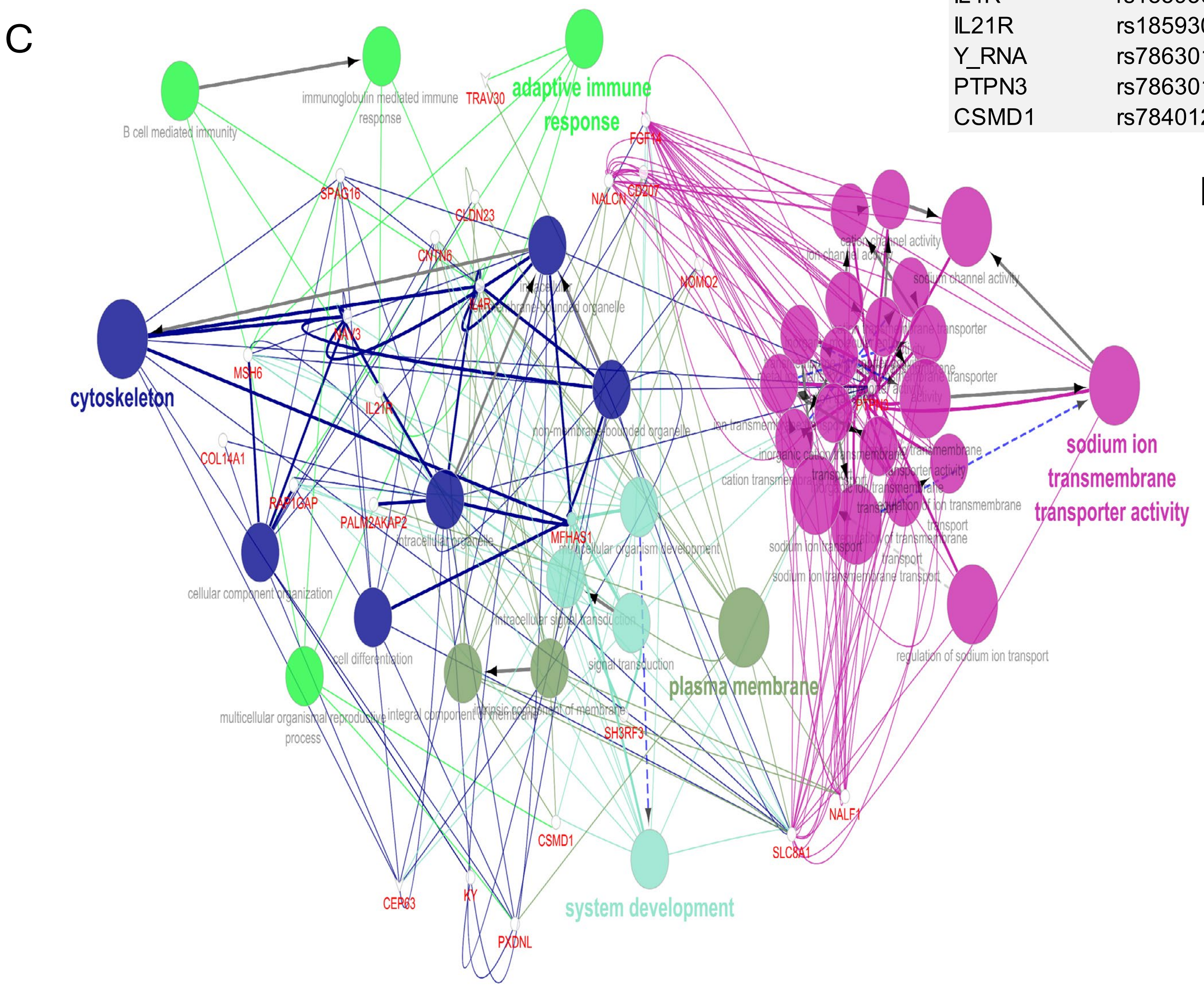

D

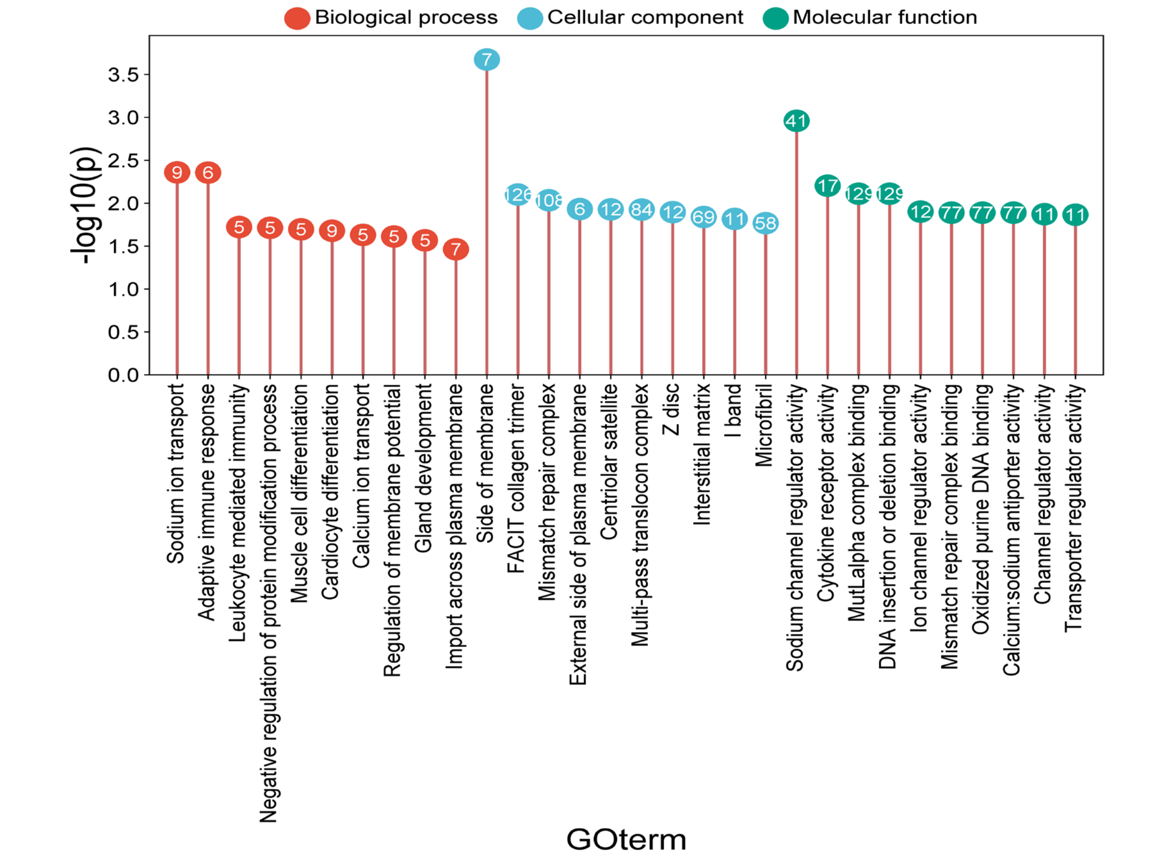

Supplement: Multimedia component 16 [file mmc16.pdf]
