# Supplementary material for: New genetic insights into HIV-associated neurocognitive disorder and Alzheimer's disease
Source: Genes Dis. 2025 Feb 26;12(5):101576. doi: 10.1016/j.gendis.2025.101576 (PMC12142519; doi:10.1016/j.gendis.2025.101576)
Supplement: Multimedia component 12 [file mmc12.pdf]

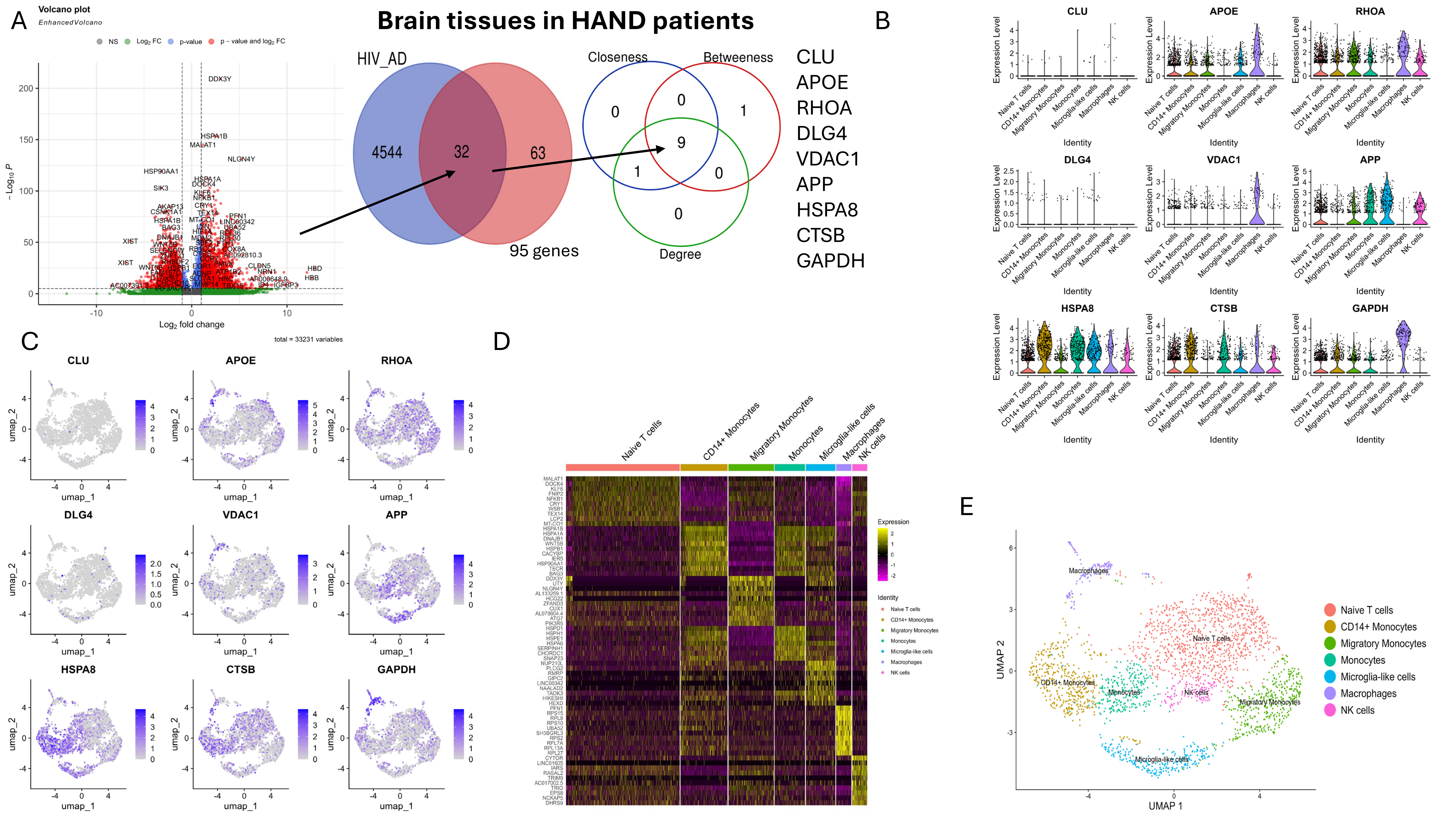
Brain tissues in HAND patients
A
Volcano plot
EnhancedVolcano
HIV_AD
4544
32
63
95 genes
Closeness
Betweeness
Degree
9
CLU
APOE
RHOA
DLG4
VDAC1
APP
HSPA8
CTSB
GAPDH
B
C
D
E
Naive T cells
CD14+ Monocytes
Migratory Monocytes
Monocytes
Microglia-like cells
Macrophages
NK cells
