# Supplementary material for: New genetic insights into HIV-associated neurocognitive disorder and Alzheimer's disease
Source: Genes Dis. 2025 Feb 26;12(5):101576. doi: 10.1016/j.gendis.2025.101576 (PMC12142519; doi:10.1016/j.gendis.2025.101576)
Supplement: Multimedia component 14 [file mmc14.pdf]

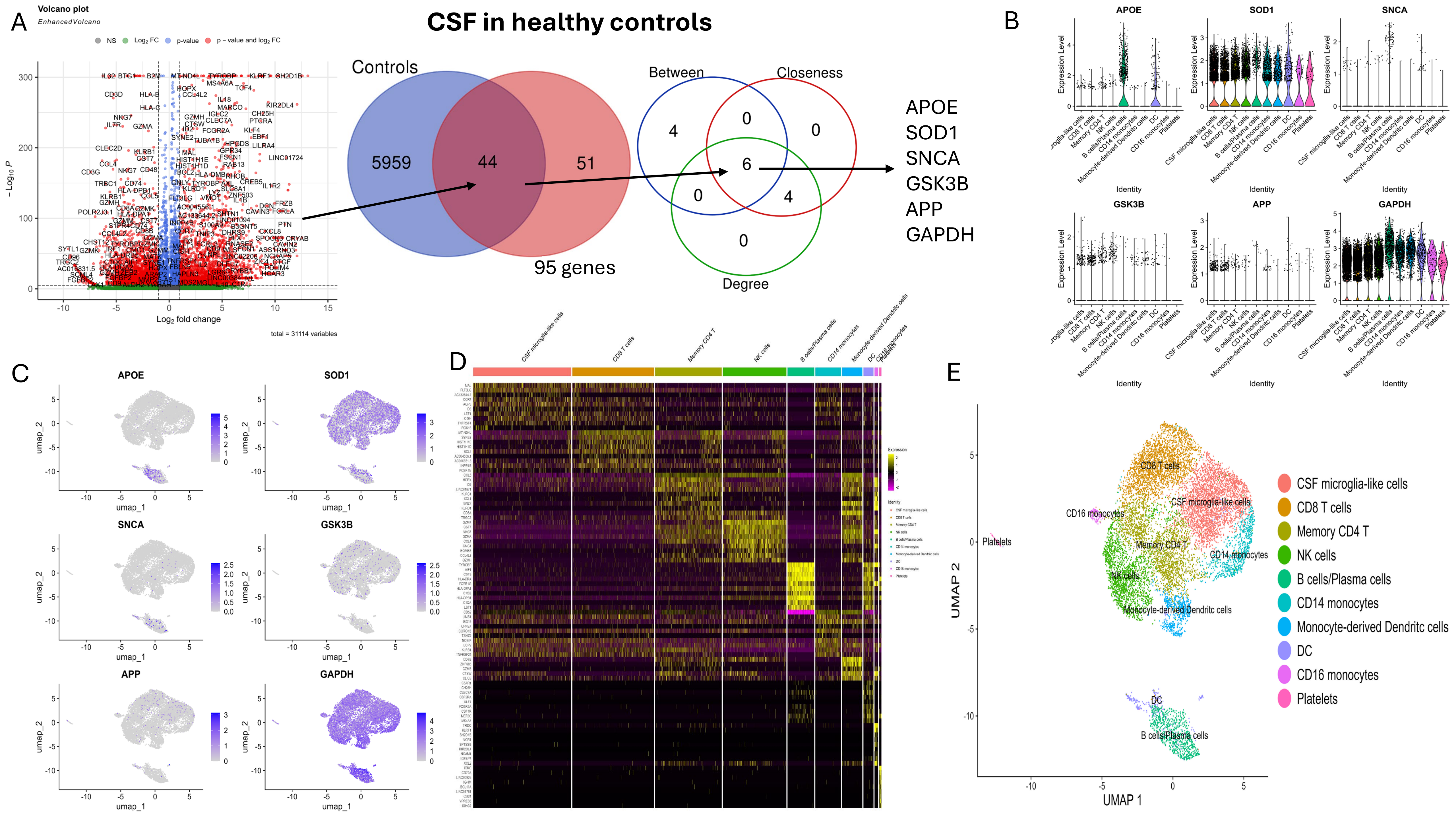
CSF in healthy controls
A
Volcano plot
EnhancedVolcano
NS
Log2 FC
p-value
p – value and log2 FC
Log2 fold change
– Log10 P
total = 31114 variables
Controls
5959
44
51
95 genes
Between
Closeness
Degree
4
0
0
6
0
4
0
APOE
SOD1
SNCA
GSK3B
APP
GAPDH
B
APOE
SOD1
SNCA
GSK3B
APP
GAPDH
Expression Level
Identity
CSF microglia-like cells
CD8 T cells
Memory CD4 T
NK cells
B cells/Plasma cells
CD14 monocytes
Monocyte-derived Dendritic cells
DC
CD16 monocytes
Platelets
C
APOE
SOD1
SNCA
GSK3B
APP
GAPDH
umap_1
umap_2
D
Expression
Identity
E
CSF microglia-like cells
CD8 T cells
Memory CD4 T
NK cells
B cells/Plasma cells
CD14 monocytes
Monocyte-derived Dendritc cells
DC
CD16 monocytes
Platelets
UMAP 1
UMAP 2
